# Prodromal neuroinflammatory, cholinergic and metabolite dysfunction detected by PET and MRS in the TgF344-AD transgenic rat model of AD: a collaborative multi-modal study

**DOI:** 10.7150/thno.56059

**Published:** 2021-05-03

**Authors:** Aisling M Chaney, Francisco R Lopez-Picon, Sophie Serrière, Rui Wang, Daniela Bochicchio, Samuel D Webb, Matthias Vandesquille, Michael K Harte, Christina Georgiadou, Catherine Lawrence, Julie Busson, Johnny Vercouillie, Clovis Tauber, Frédéric Buron, Sylvain Routier, Tristan Reekie, Anniina Snellman, Michael Kassiou, Johanna Rokka, Karen E Davies, Juha O Rinne, Dervis A Salih, Frances A Edwards, Llwyd D Orton, Stephen R Williams, Sylvie Chalon, Hervé Boutin

**Affiliations:** 1 Faculty of Biology, Medicine and Health, School of Health Sciences, Division of Informatics, Imaging and Data Sciences, University of Manchester, Manchester, UK.; 2 Wolfson Molecular Imaging Centre, University of Manchester, Manchester, UK.; 3 MediCity Research Laboratory, University of Turku, Turku, Finland.; 4 PET Preclinical Laboratory, Turku PET Centre, University of Turku, Turku, Finland.; 5 UMR 1253, iBrain, Université de Tours, Inserm, Tours, France.; 6 Department of Neuroscience, Physiology and Pharmacology, University College London, London, UK.; 7 Faculty of Biology, Medicine and Health, School of Biological Sciences, Division of Neuroscience and Experimental Psychology, University of Manchester, M13 9PL, UK.; 8 School of Healthcare Science, Department of Life Sciences, Manchester Metropolitan University, Manchester, UK.; 9 Faculty of Biology, Medicine and Health, School of Health Sciences, Division of Pharmacy and Optometry, University of Manchester, Manchester, UK.; 10 Geoffrey Jefferson Brain Research Centre, Manchester Academic Health Science Centre, Northern Care Alliance & University of Manchester, Manchester, UK.; 11 CERRP, Centre d'Etudes et de Recherches sur les Radiopharmaceutiques, Tours, France.; 12 ICOA, UMR CNRS 7311, Université d'Orléans, Orléans, France.; 13 School of Chemistry, University of Sydney, NSW, Australia.; 14 Turku PET Centre, Radiopharmaceutical Chemistry Laboratory, University of Turku, Turku, Finland.; 15 Turku PET Centre, Turku University Hospital, Turku, Finland.; 16 Division of Clinical Neurosciences, Turku University Hospital, Turku, Finland.

**Keywords:** Alzheimer's disease, animal models, positron emission tomography, magnetic resonance spectroscopy, neuroinflammation

## Abstract

Mouse models of Alzheimer's disease (AD) are valuable but do not fully recapitulate human AD pathology, such as spontaneous Tau fibril accumulation and neuronal loss, necessitating the development of new AD models. The transgenic (TG) TgF344-AD rat has been reported to develop age-dependent AD features including neuronal loss and neurofibrillary tangles, despite only expressing *APP* and *PSEN1* mutations, suggesting an improved modelling of AD hallmarks. Alterations in neuronal networks as well as learning performance and cognition tasks have been reported in this model, but none have combined a longitudinal, multimodal approach across multiple centres, which mimics the approaches commonly taken in clinical studies. We therefore aimed to further characterise the progression of AD-like pathology and cognition in the TgF344-AD rat from young-adults (6 months (m)) to mid- (12 m) and advanced-stage (18 m, 25 m) of the disease.

**Methods:** TgF344-AD rats and wild-type (WT) littermates were imaged at 6 m, 12 m and 18 m with [^18^F]DPA-714 (TSPO, neuroinflammation), [^18^F]Florbetaben (Aβ) and [^18^F]ASEM (α7-nicotinic acetylcholine receptor) and with magnetic resonance spectroscopy (MRS) and with (S)-[^18^F]THK5117 (Tau) at 15 and 25 m. Behaviour tests were also performed at 6 m, 12 m and 18 m. Immunohistochemistry (CD11b, GFAP, Aβ, NeuN, NeuroChrom) and Tau (S)-[^18^F]THK5117 autoradiography, immunohistochemistry and Western blot were also performed.

**Results:** [^18^F]DPA-714 positron emission tomography (PET) showed an increase in neuroinflammation in TG vs wildtype animals from 12 m in the hippocampus (+11%), and at the advanced-stage AD in the hippocampus (+12%), the thalamus (+11%) and frontal cortex (+14%). This finding coincided with strong increases in brain microgliosis (CD11b) and astrogliosis (GFAP) at these time-points as assessed by immunohistochemistry. *In vivo* [^18^F]ASEM PET revealed an age-dependent increase uptake in the striatum and *pallidum/nucleus basalis of Meynert* in WT only, similar to that observed with this tracer in humans, resulting in TG being significantly lower than WT by 18 m. *In vivo* [^18^F]Florbetaben PET scanning detected Aβ accumulation at 18 m, and (S)-[^18^F]THK5117 PET revealed subsequent Tau accumulation at 25m in hippocampal and cortical regions. Aβ plaques were low but detectable by immunohistochemistry from 6 m, increasing further at 12 and 18 m with Tau-positive neurons adjacent to Aβ plaques at 18 m. NeuroChrom (a pan neuronal marker) immunohistochemistry revealed a loss of neuronal staining at the Aβ plaques locations, while NeuN labelling revealed an age-dependent decrease in hippocampal neuron number in both genotypes. Behavioural assessment using the novel object recognition task revealed that both WT & TgF344-AD animals discriminated the novel from familiar object at 3 m and 6 m of age. However, low levels of exploration observed in both genotypes at later time-points resulted in neither genotype successfully completing the task. Deficits in social interaction were only observed at 3 m in the TgF344-AD animals. By *in vivo* MRS, we showed a decrease in neuronal marker N-acetyl-aspartate in the hippocampus at 18 m (-18% vs age-matched WT, and -31% vs 6 m TG) and increased Taurine in the cortex of TG (+35% vs age-matched WT, and +55% vs 6 m TG).

**Conclusions:** This multi-centre multi-modal study demonstrates, for the first time, alterations in brain metabolites, cholinergic receptors and neuroinflammation *in vivo* in this model, validated by robust *ex vivo* approaches. Our data confirm that, unlike mouse models, the TgF344-AD express Tau pathology that can be detected via PET, albeit later than by *ex vivo* techniques, and is a useful model to assess and longitudinally monitor early neurotransmission dysfunction and neuroinflammation in AD.

## Introduction

Alzheimer's disease (AD) is a major global health problem, affecting approximately 50 million people worldwide 1. Yet, our understanding of the mechanisms leading to progressive neurodegeneration and dysfunction in AD remains incomplete. Clinical AD pathology is characterised by progressive amyloid-beta plaque (Aβ) deposition, neurofibrillary tangle (NFT) formation due to Tau protein aggregation and neuronal loss. However it has become clear that AD is a complex and multi-faceted disease involving not only Aβ and Tau pathology but also chronic activation of microglia and astrocytes 2, 3, metabolic dysfunction 4, and altered cholinergic activity 5. Moreover, it is hypothesised that pathological alterations may begin many years before AD symptoms become apparent making early diagnosis and disease management challenging. Therefore, a better understanding of the pathophysiology underlying AD manifestation and progression is urgently needed to improve diagnosis and treatment of this devastating disorder.

Preclinical investigations using animal models are an essential part of the arsenal of tools to probe specific disease mechanisms and test the efficacy of therapeutic and diagnostic strategies within a living organism. Genetically modified mouse models are commonly used in AD research, allowing the investigation and confirmation of pathology induced by human familial AD mutations associated with amyloid precursor protein (APP) and presenilin 1 or 2 (PS1/PS2). These mouse models develop AD-like amyloid pathology such as age-dependent increases in Aβ plaque deposition, soluble and insoluble Aβ_40_/Aβ_42_ levels, and cerebral amyloid angiopathy (CAA) 6. Although all these transgenes-induced AD models are modelling the familial rather than the sporadic AD, until models of spontaneous AD exist, these models are beneficial in gaining a better understanding of amyloidogenic pathways and other disease processes. However, most of these models do not develop neuronal loss or NFTs without the integration of a Tau transgene. While some amyloid models have been reported to express increased hyperphosphorylated Tau, Tau aggregates similar to NFTs are not observed 7, 8. This lack of concordance with the clinical situation may be in part responsible for the low success in translatability of AD therapies. Hence, there has been a drive for improved animal models, which encompass the multiple pathological features observed in clinical AD. Consequently, transgenic AD rat models have been developed, with rats having the additional advantage over mice of a larger brain size for *in vivo* imaging and improved possibility for behavioural testing 9. Early genetic rat models were unsuccessful in precipitating extracellular amyloid plaques 10; later attempts yielded better results but most developed only Aβ plaques (for review see Do Carmo and Cuello, 2013 11). However, a more recent model, the TgF344-AD rat, described by Cohen *et al.* 12, demonstrated an age-dependent progressive amyloid pathology including plaque deposition, CAA, inter-neuronal and soluble amyloid in regions associated with and at levels comparable to that observed in clinical AD. Moreover, NFT-like Tau pathology as well as neuronal loss and neuroinflammation were also reported, overall providing a better representation of AD-like pathology. In their study Cohen *et al.* characterised the model at 6, 16 and 26 months (m) of age. Increased Aβ_40_ was reported as early as 6 m with cognitive deficits by 15 m and NFT deposition by 16 m. Finally, increased microglial and astrocyte activation were detected *ex vivo* as early as 6 m of age. Since then, various deficits and dysfunctions have been reported at different ages in these rats including alterations in hippocampal and cortical neurotransmission 13-16, neurovascular dysfunction 17, behavioural deficits 18-21, functional connectivity alterations 22-24 and blood-brain barrier alterations 25. Hence this model seems to be a good candidate, yet some important parameters have not been investigated and questions remain as to whether this is a useful model to investigate longitudinal* in vivo* markers of AD.

Here we take an *in vivo* multi-modal approach to characterise AD development and progression in TgF344-AD and wildtype (WT) rats from early to more advanced stages of the disease (6 to 25 m), including non-invasive longitudinal imaging, *ex vivo* immunohistochemical analysis and cognitive assessments. Specifically, and for the first time, [^18^F]DPA-714, [^18^F]ASEM, [^18^F]Florbetaben ([^18^F]AV-1) and (S)-[^18^F]THK5117 PET imaging were performed to assess neuroinflammation, the acetylcholine system, Aβ plaque deposition and Tau aggregates respectively. Additionally, magnetic resonance spectroscopy (MRS) was performed to assess metabolite alterations associated with neuronal dysfunction and inflammation. Altogether, those *in vivo* measurements, combined with *ex vivo* immunohistochemical assessments are providing novel and wide-ranging information about the neuropathological characteristics and utility of this model.

## Materials and Methods

### Animals

Two male and two female WT Fischer and TgF344-AD rats with the APP_swe_ and PS1_Δe9_ mutations were purchased from the laboratory of Prof T. Town (University of Southern California) and were set up as breeding pairs, housed in the Biological Services Unit at the University of Manchester. Genotyping was outsourced to Transnetyx®. All animals were housed in groups of 2-4 per cage with individual ventilation, environmental enrichment, constant access to food and water and a 12:12 hour cycle of light and dark.

*In vivo* experiments were conducted across three centres: University of Manchester (UK), University of Tours (France) and University of Turku (Finland) and animals were housed and fed in the same conditions as in Manchester. All experiments carried out at the University of Manchester were performed in accordance with the Animal Scientific Procedures Act 1986, following internal ethical review. Breeding pairs from this colony were sent to the University of Tours and University of Turku where rats were bred for investigation of amyloid ([^18^F]Florbetaben), α7-nAchR ([^18^F]ASEM) and Tau ((S)-[^18^F]THK5117). In Tours, animals were treated in accordance with the European Community Council Directive 2010/63/EU for laboratory animal care and the experimental protocol was validated by the Regional Ethical Committee (Authorization n°4795.03). Genotyping was outsourced to Charles River GEMS (France). In Turku all animal experiments were approved by the Regional State Administrative Agency for Southern Finland (ESAVI/3899/04.10.07/2013 and ESAVI/4499/04.10.07/2016), and animal care complied with the principles of the laboratory animal care and with the guidelines of the International Council of Laboratory Animal Science.

Details of numbers of animals for each experiment are provided below and in Tables S1-S4. Survival curves and mortality/exclusions are shown for the University of Manchester cohort in Table S4 and Figure S1.

### Study Design

Animals bred at the University of Manchester underwent behavioural testing, MRS, [^18^F]DPA-714 PET, and immunohistochemistry studies. N number per group were based on power analysis of our own or previously published data with α = 0.05 and β = 0.95 12, 21, 26-30. Various parameters including moderate increases in neuroinflammation, Aβ and Tau have been reported in the Tg-F344-AD rat via immunohistochemistry at 6, 16 and 26 m 12. Thus, [^18^F]DPA-714 PET was performed at 6 m, 12 m and 18 m to non-invasively assess longitudinal alterations in neuroinflammation in WT and TG rats from early to advanced disease. Similarly, these time-points were used for MRS imaging and behavioural assessment. In particular, the 12 m time-point was chosen because we wanted to address the progression in the critical prodromal stage of the disease and from Cohen *et al.* 12 it seemed clear that AD-like pathology was already quite advanced by 16 m of age. Brain tissue was harvested in a subset of rats at each time-point (Table S1) for *ex vivo* analysis *i)* between 5m 3 weeks and 7m 2 weeks (referred to as the 6 m time-point; WT: 208 ± 24 days, n = 5 (mean ± SD); TG: 203 ± 21 days, n = 8),* ii)* between 11m 3 weeks and 13 m 2 weeks (12 m time-point; WT: 391 ± 23 days, n = 5; TG: 389 ± 16 days, n = 6) and *iii)* between 18 m 2 weeks and 19m 3 weeks (18 m time-points; end-point for animals from the longitudinal study and few additional animals; WT: 585 ± 11 days, n = 13; TG: 591 ± 14 days, n = 11) (See Tables S1 and S2 for full details). There was a minimum gap of one week between anaesthetising animals for imaging and conducting behavioural tests. Imaging experiments were carried out during the light phase of day. Behavioural assessments begun at the end of the dark phase (4-7 AM) as the rats were more active and exploratory behaviour was increased at this time.

WT and TG males (n = 8 per group) bred at the University of Tours were enrolled in [^18^F]Florbetaben and [^18^F]ASEM PET studies at 6, 12 and 18 m (for more details see Table S3).

Tau pathology was assessed in 3 WT and 4 (one animal died at 24 months of age) TG females bred at the University of Turku using (S)-[^18^F]THK5117 PET at 15 and 25 m and *ex vivo* autoradiography at 25m. Tau studies were conducted at later time-points owing to previous findings indicating NFT-like Tau deposition in these rats from a mid-disease state (i.e., 16 m) 12. At 15 m (457 ± 9 days) rat weights were WT 295 ± 5g and TG 302 ± 28g. At 25 m (748 ± 11 days) rat weights were WT 339 ± 37g and TG 340 ± 6g.

### PET Acquisition and Analysis

#### [^18^F]DPA-714 PET

To assess *in vivo* neuroinflammatory status [^18^F]DPA-714 PET was performed in WT and TG rats at 6, 12 and 18 m. [^18^F]DPA-714 was synthesized as previously described 31. Animals underwent anaesthesia (1.5-2.5% isoflurane in a 30%/70% 0_2_/NO_2_ mixture at 1.2 l/min), tail vein cannulation, [^18^F]DPA-714 injection (33.1 ± 6.8 MBq) and 60 min dynamic PET/CT imaging. Imaging was carried out on a Siemens Inveon® PET/CT scanner and image reconstruction was performed as previously described 26, 27 (see Suppl. Material for more details). Skeletal and brain regions were defined manually in the CT images using Brainvisa and Anatomist software (http://brainvisa.info) to register PET-CT images with a MRI template; ROIs were quantified in Brainvisa by applying a rat MRI template adapted from Schwarz and colleagues 32. Co-registration with a MRI atlas seem appropriate as Anckaerts *et al.* 22 did not report any age-dependent genotype-specific cortical atrophy or ventricular enlargement in the TG groups. ROIs included: entorhinal cortex, cingulate cortex, frontoparietal motor and somatosensory cortex, frontal cortex, temporal cortex, hippocampus, thalamus, hypothalamus and striatum. For the PET quantification the summed frames from 20 to 60 (Figure S3A) min were used to calculate the uptake values normalised to cerebellum (NUV_Cb_). The cerebellum was chosen as it is routinely used (clinically and preclinically) as a pseudo-reference region for TSPO-PET quantification in Alzheimer's disease populations 26, 29, 33-36.

#### [^18^F]Florbetaben and [^18^F]ASEM PET

[^18^F]florbetaben and [^18^F]ASEM PET were performed to assess respectively amyloid burden and α7 nicotinic acetylcholine receptor (α7-nAchR) density in WT and TG rats at 6, 12 and 18 m (see Table S3). [^18^F]Florbetaben 37 and [^18^F]ASEM 30 were synthesized as described previously. Anaesthetised animals (1.5-2% isoflurane in 1.5-2 L/min of O_2_) were scanned over 51 min for [^18^F]Florbetaben and 61 min for [^18^F]ASEM on a superArgus PET/CT system (Sedecal, Spain) according to Sérrière *et al*. 29 (for more details see Suppl. Material). Before PET acquisition, a 5 min CT scan was acquired for attenuation correction. Animals received a bolus injection of 37 MBq/350 g body weight of [^18^F]Florbetaben or [^18^F]ASEM in a saline solution via the tail vein. Images were analysed using PMOD (3.403, PMOD Technologies, Zurich, Switzerland). Partial volume effect correction was applied on all PET images which were co-registered to the Schiffer rat brain MRI template for regions of interest (ROI) analysis. Normalised uptake values (NUV) were calculated using the brainstem as reference region for [^18^F]Florbetaben (last 3 frames, 43-51 min acquisition, Figure S3B) 38 and cerebellum for [^18^F]ASEM 39 (last 6 frames, 49-61 min acquisition, Figure S3C).

#### (S)-[^18^F]THK5117 PET and autoradiography

To assess Tau deposition, (S)-[^18^F]THK5117 PET was performed in WT and TG rats at 15 and 25m. (S)-[^18^F]THK5117 was synthesized as previously described 40. Prior to PET acquisition, a CT scan was acquired for attenuation correction. The rats were anesthetised with 2.5% isoflurane in a 30%/70% O_2_/NO_2_ mixture at 400-500 mL/min and then injected with a bolus injection of (S)-[^18^F]THK5117 (23.7 ± 3.0 MBq) and 60 min dynamic scans were acquired using a Siemens Inveon® PET/CT scanner and reconstructed with OSEM-3D. PET images were pre-processed and co-registered to the Schiffer rat brain MRI template for ROI analysis (for more details see Suppl. Material). Following the final *in vivo* scan, the brains were quickly removed and frozen in isopentane (2-methylbutane; Sigma-Aldrich) on dry ice to perform *ex vivo* autoradiography studies. Coronal sections (20 μm) were obtained using a cryomicrotome (Leica CM3050S) and collected on a glass slide (Superfrost Ultra Plus; Thermo Fisher). The slides were exposed to an image plate (Fuji BAS Imaging Plate TR2025; Fuji Photo Film Co., Ltd.) for 4 hours.

For the PET quantification the summed frames from 30 to 40 min were used (Figure S3D); PET and autoradiography data are expressed as standard uptake values normalised to striatum (NUV_str_). ROIs included: temporal-auditory, piriform, frontal, frontoparietal and cingulate cortices, hippocampus, thalamus, pons, cerebellum, hypothalamus and striatum. PET and autoradiography quantification were performed as previously described 41.

### Magnetic Resonance Spectroscopy Acquisition and Analysis

To assess metabolite profile, single voxel MRS was performed in the full hippocampus (2 × 9 × 3 mm^3^), right hippocampus (2 × 4.5 × 3 mm^3^), thalamus (2.5 × 8 × 3 mm^3^), hypothalamus (2 × 3 × 3 mm^3^) and cortex (1.2 × 3 × 3 mm^3^) in TG and WT rats at 6, 12 and 18 m of age (for localisation of the voxel for each brain region, see Figure S2). Isoflurane (2-3%) in oxygen (2L/min) was used to anesthetise and maintain rats. MRI and MRS were performed and analysed as previously described 26, 42 (see Suppl. Material for more details). Respiratory rate (65-80 breaths per min) and temperature (36.5-37 °C) were monitored and maintained throughout by altering respectively the anaesthesia and hot air supply. Metabolites are expressed as amplitude of institutional units relative to water.

### Immunohistochemistry

For full details of Immunohistochemistry protocols, please see Suppl. Material. For all immunohistochemical analysis, the observers were blinded to the genotype and age-group of the animals, having only access to the animal number given at birth to each animal until all images were captured and analysed. However, it must be noted that the presence of Aβ plaques and associated microglial activation and astrogliosis were obvious when looking at the brain sections under a microscope. Number of animals used for each quantitative immunohistochemistry can be found in Table S5.

#### Immunofluorescence for GFAP & CD11b, NeuN, Neurochrom and amyloid

Animals were culled and brain were processed and cut as described previously 26, 27 (see Suppl. Material for more details).

Immunohistochemistry was carried out on TG and WT 20 µm thick frozen brain sections (n = 5-9 per group, see Table S5) to visualise CD11b (microglial marker), GFAP (astrocytic marker), NeuN and Neurochrom (neuronal markers), and 6E10 (Aβ marker) as previously described 26 (for more details see Suppl. Material).

Three to five images from 2-4 brain sections spanning between 1 and 2.86 mm lateral of Bregma were collected for each brain structure (hippocampus, thalamus and cortex) per animal. Image were collected on an Olympus BX51 upright microscope using 10×/0.30 or 20×/0.50 UPlanFLN objectives and captured using a Retiga 6000 Color camera through QCapture Pro 7 Software (QImaging Inc.). Specific band pass filter sets were used to prevent bleed through from one channel to the next.

6E10 slides were scanned using a 3D Histec Pannoramic250 slide scanner and 1-4 snapshots per brain area of interest were taken using 3D Histec CaseViewer software. All snapshots were analysed using Fiji 43 (for full details see Suppl. Material).

#### Chromogenic NeuN immunohistochemistry and analysis

Sagittal sections were stained with rabbit anti-NeuN and Vector DAB HRP substrate (For full details of protocol see Suppl. Material).

#### Amyloid Thioflavin-S staining and Tau immunohistochemistry

Because our attempt to perform immunohistochemistry for Tau using snap-frozen brain sections failed, we looked for PFA-perfused-fixed brains from one of our partners of the INMiND consortium. Perfused-fixed brains from 18 m old animals were a generous gift from Dr Guadalupe Soria (IDIBAPS, Barcelona, Spain) and were used for Phospho-Tau immunohistochemistry and amyloid Thioflavin-S staining. PFA-perfused-fixed brains were cryoprotected in 30% sucrose, sectioned into 30 µm thick sagittal sections using a Leica frozen microtome and stored as free-floating brain sections at 4 °C in PBS/0.3% azide. Phospho-Tau and Thioflavin-S co-staining: sections were permeabilised by incubating with 0.3% Triton-X 100/PBS then endogenous peroxidase was quenched in 0.5% H2O2 for 30 min, and blocked in 2.5% normal horse serum (MP-7422, Vector Laboratory) followed by incubation with AT8, CP13 or PHF-1 primary antibody overnight at 4°C. Secondary Anti-Mouse IgG (MP-7422, Vector Laboratory) was applied for 30 min, followed by peroxidase substrate solution (SK-4105, Vector Laboratory) until desirable stain intensity developed. Sections were then incubated in 1% Thioflavin-S (T1892, SIGMA) solution for 7 min and washed with 70% Ethanol, before mounting with Fluoromount-G medium (Southern Biotech).

The entire hippocampus was imaged in each section using an EVOS FL Auto microscope (Life Technologies) with a ×20 objective, by area defined serial scanning (n = 3 per genotype). The image was processed using EVOS FL Auto Cell Imaging System and Adobe Photoshop CS6.

### Tau Western blot analysis

Approximately 100 mg of cortex tissue was homogenised in 1 mL cold RIPA buffer as described previously 44. The protein sample for Tau expression analysis was extracted from cortical tissue homogenate then lysed in Tau Dye. Lysed protein was boiled for 5 min at 95 °C. Next, Western blotting was performed using the SDS-PAGE gel and nitrocellulose membrane, with 50 µg protein loaded for each sample. CP13, PHF-1, DA9 and HSPA9 primary antibodies were used (for more details see Table S6). Protein bands were visualized using enhanced chemiluminescence. Imaging was performed using the BIO-RAD ChemiDoc MP imaging system and protein expression bands were analysed by Image Lab software (BIO-RAD) (for more details see Suppl. Material).

### Behaviour

The background strain (Fischer 344) used to generate the TgF344-AD has been reported to be highly anxious and proved to be so in our hands (avoidance, agitated when handled, vocalising, biting) 45, 46. We thus ensured that animals were extensively handled prior to behavioural testing (see details in Suppl. Material).

#### Novel Object Recognition Test

To assess short-term non-associative working memory, a novel object recognition (NOR) test was carried out in WT (n = 9-10) and TG (n = 9-11; see Table S1 for details) rats at 3, 6, 12 and 18 m as previously described 47. In brief, NOR tests were performed in 3% light, and involved acquisition, delay and retention phases of 3 min each. Time spent exploring the novel and familiar objects in the retention phase was used to quantify the discrimination index (DI) for each animal, defined as the novel minus the familiar time divided by the total time, giving values ranging from -1 to +1. Animals were excluded from analysis if side bias was displayed in the acquisition phase (> 60% time on one side) or if they did not demonstrate exploratory behaviour towards any objects in the retention phase (< 2s cut-off; 3 m: 2 TG; 6 m: 2 WT and 1 TG; 12 m: 2 WT and 3 TG; and 18 m: 3 WT and 1 TG were excluded).

#### Social Interaction Test

A social interaction test was used to assess anxiety-like behaviour 48 in the WT (n = 9) and TG rats (n = 9-10; see Table S1 for details) at 9, 12, 15 and 18 m. This test was carried out in 45% light. All rats were age and weight matched to avoid/minimise dominance and fighting behaviour. In brief, a test rat and an unfamiliar wildtype conspecific rat were placed in an arena for 10 min with an inanimate object (e.g. metal can or plastic bottle) in the centre. Time spent sniffing, following, and avoiding the conspecific animal as well as exploring the central object were quantified and data expressed as a discrimination index (see above). Arenas were cleaned with 70% ethanol in between trials.

### Statistical analysis

The data were statistically analysed using GraphPad® Prism™ (v8.4, GraphPad Software, Inc., San Diego, California USA).

Shapiro-Wilk normality test was carried out for raw exploration times to assess side bias and, depending on the normality of the distribution, either *t*-tests or Wilcoxon tests were used. Survival rate of the University of Manchester cohort was analysed using a Log-rank Mantel-Cox test. Mixed model effects analysis was used to assess alterations in DI in the NOR test. T-tests were used to investigate time spent exploring familiar and novel objects as well as social interaction behaviours in WT and TG animals at individual time-points. One-way ANOVA was used to assess levels of exploration over time in each group.

Mixed model effects analysis was used to assess the effect of genotype and age (as repeated factor) and possible interaction on MRS metabolites levels and [^18^F]DPA-714 and (S)-[^18^F]THK5117 PET NUV values in WT and TG. [^18^F]ASEM PET NUV were analysed using 2-way ANOVA to assess the effect of genotype and age (as repeated factor) and possible interaction.

Normality of the CD11b, GFAP, NeuN and Aβ immunohistochemistry quantitative data was analysed using d'Agostino and Pearson test and if significant, outliers were removed. Only GFAP in the temporal/posterior cingulate cortex and CD11b and GFAP in the thalamus did not pass the normality test and outliers were removed (see Table S5). Immunohistochemistry quantitative data were analysed using 2 way-ANOVA (genotype and age).

If a significant effect and/or interaction were found with the mixed model effects analysis or ANOVA, a *post-hoc* Sidak test was performed to determine group differences.

Autoradiographic data for (S)-[^18^F]THK5117 comparing WT and TG at 25m of age were analysed with Welsh's *t-*tests. For all statistical analyses, the significance level was p < 0.05.

### Data availability

The datasets generated during and/or analysed during the current study are available upon reasonable request. Request should be addressed to the corresponding author and data will be made available by the institution where the experiments took place.

## Results

### Animals

The study of the University of Manchester cohort revealed that the TgF344-AD strain can easily be aged up to 18-19m but that, upon reaching this age, the rate of spontaneous illnesses or deaths start to increase due to spontaneous stroke, intracerebral or subarachnoid haemorrhage or tumours (Figure S1 and Tables S1 and S4). The survival rate was not however significantly associated with genotype (p = 0.2748, Log-rank Mantel-Cox test; Figure S1). There was no statistical difference in body weights between the 2 cohorts of male rats (Tours and Manchester) presented in Table S2 and S3 (data not shown).

### Neuroinflammation and reactive gliosis detected by [^18^F]DPA-714 PET and immunohistochemistry are increased in the hippocampus, cortex and thalamus of aged Tg-F344-AD rats

**Figure 1:**
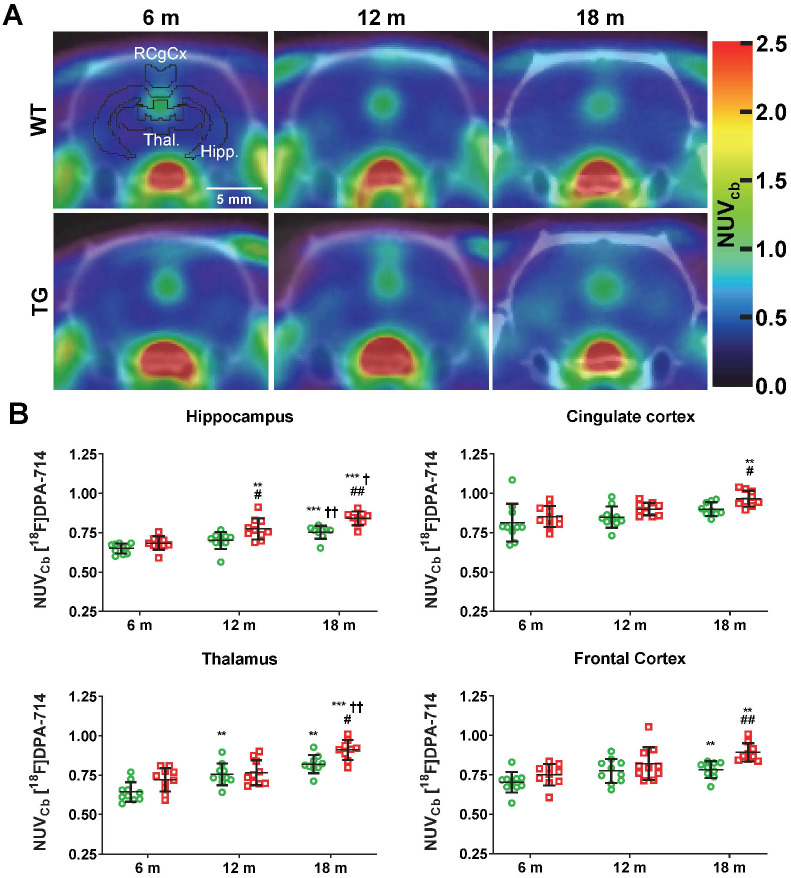
(**A**) Representative averaged 20-60 min [^18^F]DPA-714 PET-CT images co-registered with the MR template. ROIs for the retrosplenial & cingulate cortices (RCgCx), thalamus (Thal) and hippocampus (Hipp) are shown in the top left (6 m, WT) image. The frontal cortex is not shown on the PET-CT images as this region is more rostral. (**B**) [^18^F]DPA-714 uptake quantification in various regions of the brain in WT (green circle symbols) and TgF344-AD (red square symbols) rats at 6, 12 and 18 months (m) of age. Data are expressed as mean ± SD of uptake values normalised to cerebellum. * and ✝ indicate significant differences vs the 6 and 12 m old animals respectively and # indicates significant difference between WT and TG for rats of same age. *, ✝ or # indicates p < 0.05, **/……/## p < 0.01 and ***/………/### p < 0.001. PET data were analysed using a mixed model (age as repeated factor and genotype) and a Sidak post-hoc test.

[^18^F]DPA-714 PET was performed to non-invasively assess longitudinal alterations in neuroinflammation in WT and TG rats at 6 m, 12 m and 18 m (Figure 1). Data are presented as NUV_CB_ as the uptake in cerebellum was not affected by genotype (Figure S4A). Both genotype and age significantly affected uptake in the hippocampus (genotype p = 0.001, age p < 0.001), frontal cortex (genotype p = 0.010, age p < 0.001), thalamus (genotype; p = 0.002, age; p < 0.001) and retrosplenial/cingulate cortices (genotype p = 0.007, age p = 0.002). In the hippocampus, elevated uptake was observed in TG vs WT rats at 12 m (+11 ± 8% p = 0.045) and 18 m (+12 ± 5% p = 0.001) (Figure 1B). Additionally, increased uptake with age was seen in TG rats from 12 m (vs 6 m +13 ± 10% p = 0.008, 18 m vs 6 m +21 ± 7% p < 0.001, 18 m vs 12 m +8 ± 9% p = 0.042) but only at 18 m in WT (vs 6 m +16 ± 7% p < 0.001 and vs 12 m +7 ± 5% p = 0.001) indicating that hippocampal inflammation associated with normal aging is exacerbated in TG rats (Figure 1B). Similarly, [^18^F]DPA-714 uptake was significantly increased in TG when compared to WT rats at 18 m in the frontal cortex (+14 ± 7% p = 0.002), thalamus (+11 ± 7% p = 0.020) and retrosplenial/cingulate cortices (+7 ± 5% p = 0.024). ^18^F]DPA-714 signal was also increased with age in the retrosplenial/cingulate cortices (+13 ± 9% 18 m vs 6 m, p = 0.003) in the TG group only (Figure 1B). Moreover, [^18^F]DPA-714 uptake was only affected by genotype in the entorhinal and frontoparietal motor cortices (p = 0.042 and p = 0.046 respectively, Figure S4B).

Additionally, an effect of age was also observed in both WT and TG in some of these regions. [^18^F]DPA-714 uptake was increased with age in the frontal cortex (WT 18 m vs 6 m: +12 ± 7% p = 0.003, TG 18 m vs 6 m: +17 ± 12% p = 0.003) and thalamus (WT 12 m vs 6 m: +18 ± 15%, p = 0.009, WT 18 m vs 6 m: +30 ± 16%, p = 0.001; TG 18 m vs 6 m: +24 ± 8% p < 0.001, TG 18 m vs 12 m: +19 ± 14% p = 0.005) (Figure 1B), indicating that these region are also affected by normal aging.

In contrast, in temporal-auditory cortex, frontoparietal somatosensory cortex and striatum, a significant effect of age only was detected (p = 0.042, p < 0.001, p < 0.001 respectively) (Figure S4C). However, in the temporal-auditory cortex, this effect of age was most likely driven by the TG data with a trend for genotype effect (p = 0.0599) and a significant difference between WT and TG at 18 m (Figure S4C). In the striatum, an age effect was seen in both WT and TG with a significant increase in neuroinflammation at 12 m (+10-12%) and 18 m (+15-18%) (Figure S4C). In the hypothalamus, a significant interaction age × genotype (p = 0.01) was observed with a transient increase in [^18^F]DPA-714 uptake in TG only observed at 12 m (+26 ± 18% vs 6 m, p = 0.007 and +24 ± 21% vs 18 m p = 0.049) and returning to baseline at 18 m (Figure S4D).

**Figure 2:**
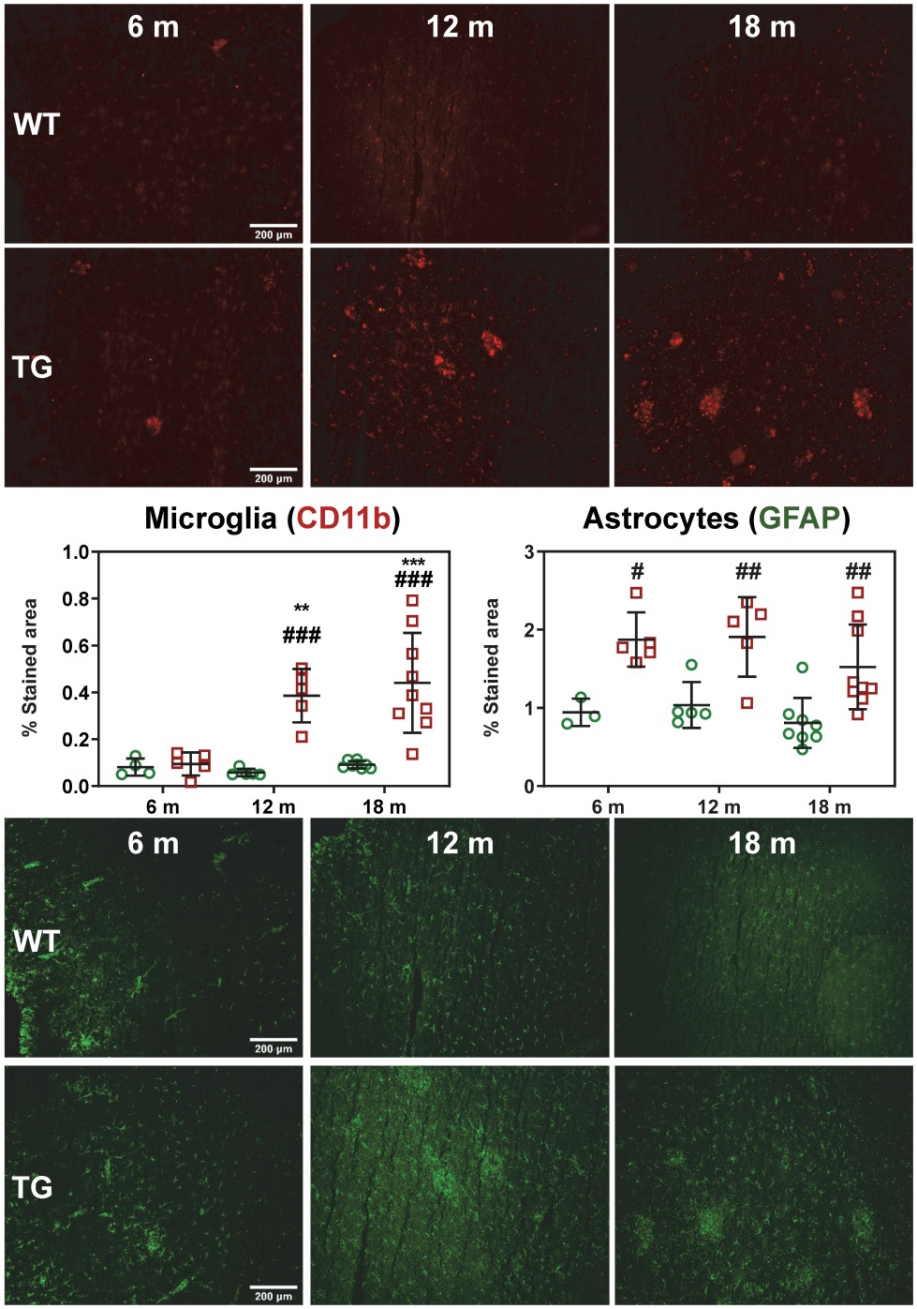
Representative micrographs of microglial (CD11b, top panel) and astrocytes (GFAP, bottom panel) in the temporal/posterior cingulate cortices of WT and TG rats at 6, 12 and 18 months (m) of age. Quantification of the immunofluorescence expressed as percentage of area stained. * and ✝ indicate significant differences vs the 6 and 12 m animals respectively and # indicates significant difference between WT and TG for rats of same age. *, ✝ or # indicates p < 0.05, **/……/## p < 0.01 and ***/………/### p < 0.001. Data were analysed using 2-way ANOVA (age as repeated factor and genotype) and a Sidak post-hoc test. Scale bars represent 200µm.

Activated microglial and astrogliosis were quantified by CD11b and GFAP immunohistochemistry labelling density at 6, 12 and 18 m (Figure 2). Quantification of images revealed both age and genotype dependent increases in CD11b-positive cells in hippocampus (genotype p < 0.001, age p = 0.002, genotype × age interaction p = 0.006), frontal cortex (genotype p < 0.001, age p < 0.003, genotype × age interaction p = 0.007), temporal cortex (genotype p < 0.001, age p = 0.004, genotype × age interaction p = 0.005) and thalamus (genotype p < 0.001, age p = 0.003). Markedly elevated CD11b staining (%stained area) was observed in TG rats vs WT in the temporal/cingulate cortices at 12 m and 18 m (+564 ± 28% and +383 ± 33% respectively, both p < 0.001, Figure 2), and at 18 m in the hippocampus (+723 ± 44% p < 0.001), frontal cortex (+435 ± 54% p < 0.001) and thalamus (+115 ± 30% p = 0.001) (Figure S5). Consistent with these results, increases in CD11b staining with age was also observed in TG rats in these regions which was not evident in the WT rats (Figure 2, Figure S5).

Astrogliosis (GFAP+) was found to be elevated in temporal/posterior cingulate (TG vs WT: 6 m: +99 ± 18%, p = 0.013; 12 m: +84 ± 27% p = 0.007; 18 m: +89 ± 38%, p = 0.004; Figure 2) and frontal cortex (TG vs WT 12 m: +109 ± 25% and 18 m: +98 ± 38% p < 0.001 for both, Figure S5). In the thalamus, astrogliosis was increased in TG vs WT at 6 m (+158 ± 21%, p = 0.001) but decreased with age to reach similar levels as in WT (6 m vs 12 m: -44 ± 42%, p = 0.008, and 6 m vs 18 m: -39 ± 32%, p = 0.007, respectively, Figure S5). In the hippocampus, there was a significant effect of genotype (p = 0.008) and a significant decrease with age at 18 m vs. 6 m in both WT (-47 ± 33%, p = 0.036) and TG (-36 ± 24%, p = 0.014, Figure S5).

### [^18^F]ASEM PET imaging indicates reduced subcortical α7-nAChR density in TgF344-AD rats

**Figure 3:**
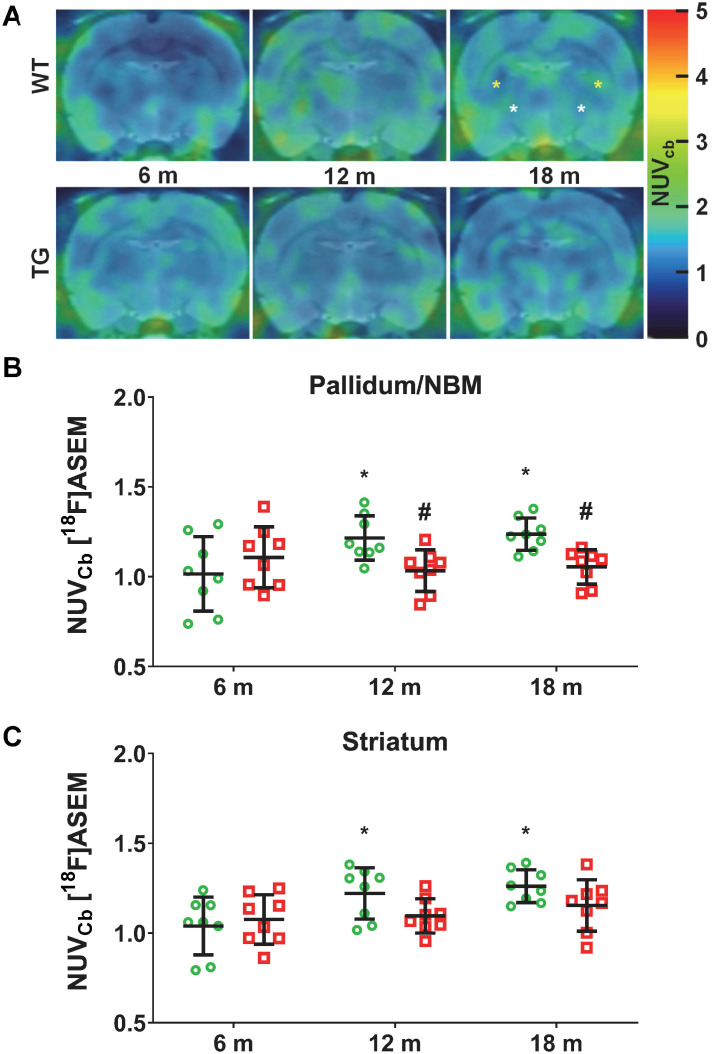
** (A)** Representative sum images (49-61 min) of [^18^F]-ASEM (α7 nicotinic receptor) PET-MR in the brain of WT and TgF344-AD rats at 6, 12 and 18 months (m) of age (*pallidum/nucleus basalis of Meynert (NBM*), striatum are respectively indicated with white, yellow * on the top right (WT 18 m) PET image). Quantification of the [^18^F]-ASEM uptake in *pallidum/NBM* (**B**) and striatum (**C**) of WT (green circle symbols) and TgF344-AD (red square symbols) rats. (**B, C**) Data are expressed as mean ± SD of uptake values (sum image 20-60 min) normalised to cerebellum. * and ✝ indicate significant differences vs the 6 and 12 m old animals respectively and # indicates significant difference between WT and TG within age groups. *, ✝ or # indicates p < 0.05. PET data were analysed using a 2-way ANOVA (age as repeated factor and genotype) and a Sidak post-hoc test.

Serial [^18^F]ASEM PET imaging was performed to assess α7-nAChR density as a marker of cholinergic function with disease progression in TG compared to WT rats. Explorations of α7-nAChRs has not previously been investigated in this model, thus [^18^F]ASEM PET was also performed at 6 m, 12 m and 18 m (Figure 3A). Significant changes in [^18^F]ASEM signal were observed in the *pallidum/nucleus basalis of Meynert (NBM)*, with a significant effect of genotype (p = 0.028) and an age × genotype interaction (p = 0.017). Increased [^18^F]ASEM uptake was observed in this brain region with age in WT rats (12 m vs 6 m: +25 ± 33%, p = 0.031; 18 m vs 6 m: +27 ± 30%, p = 0.015), which did not occur in TG, resulting in the TG being significantly lower than the WT at 12 m (-15 ± 11% , p = 0.038) and 18 m (-15 ± 8%, p = 0.038) (Figure 3B). A similar effect was detected in the striatum (age effect p = 0.019) driven by a significant increase in tracer uptake only in WT animals (12 vs 6 m: +22 ± 32%, p = 0.049; 18 m vs 6 m: +25 ± 29%, p = 0.012) (Figure 3C). There was a more modest increase with age in thalamus (age effect, p = 0.047; post-hoc 18 m vs 6 m: +10 ± 12% p = 0.045) (Figure S6A). In contrast, no significant differences were observed in cortex (Figure S6B) or hippocampus (Figure S6C-D).

### Amyloid deposition detected by [^18^F]Florbetaben PET and immunohistochemistry is increased in hippocampus and cortex of TgF344-AD rats

**Figure 4:**
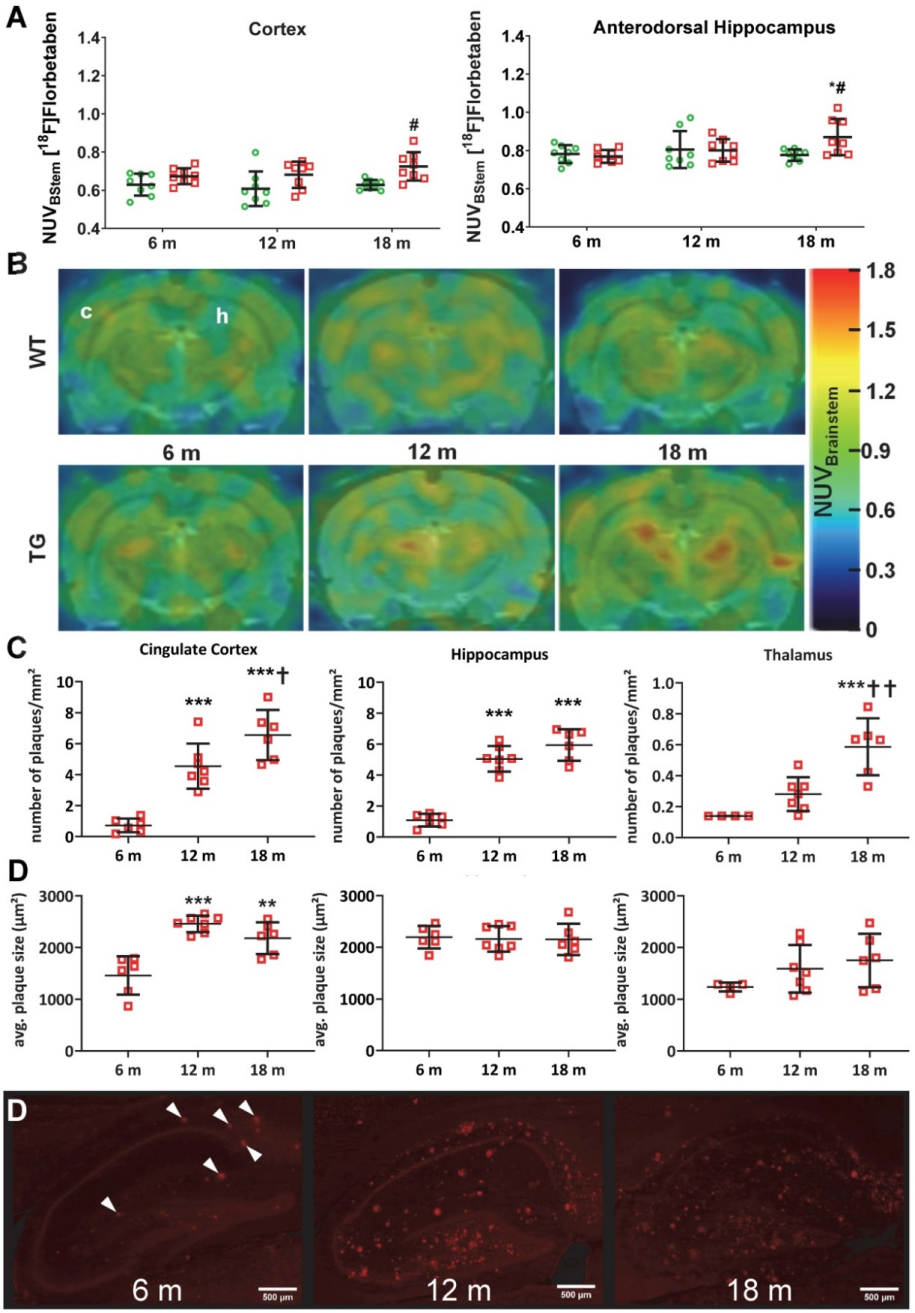
PET quantification (**A**) of [^18^F]Florbetaben uptake normalised to brain stem in the cortex and dorsal hippocampus of WT (green circle) and TG (red squares) rats and (**B**) representative PET-MR sum images (43-51 min) of [^18^F] Florbetaben brain uptake at 6, 12 and 18 months of age (In top left image, **c** and **h** denotes the position of the cortex and hippocampus respectively). (**C**) Quantification of the β-amyloid immunohistochemistry (6E10) in the cingulate cortex, hippocampus and thalamus of TG rats 6, 12 and 18 months (m) of age. NOTE there are two (D) on figure. (**D**) Representative images of the β-amyloid immunohistochemistry (6E10) in the hippocampus of TG rats (scale bars represent 500 µm). Data are expressed as mean ± SD. * and ✝ indicate significant differences vs the 6and 12 mold animals respectively and # indicates significant difference between WT and TG. *, ✝ or # indicates p < 0.05, **/……/## p < 0.01 and ***/………/### p < 0.001. PET data were analysed using a mixed model, immunohistochemistry data were analysed using a 2-way ANOVA with age as repeated factor and genotype in both analysis and a Sidak post-hoc test.

[^18^F]Florbetaben ([^18^F]AV-1) PET was conducted to assess levels of amyloid pathology in both genotypes at 6 m, 12 m and 18 m (Figure 4A-B). Analysis revealed a significant genotype effect in the cortex (p = 0.001) and genotype × age interaction in the dorsal hippocampus (p = 0.028), which resulted in increased uptake in TG compared to WT rats at 18 m in both regions (+15 ± 7% p = 0.012 and +12 ± 7% p = 0.020 respectively) (Figure 4A-B). Increased tracer uptake was also observed at 18 m vs. 6 m in TG dorsal hippocampus (+13 ± 12% p = 0.005). Progressive amyloid burden was confirmed via 6E10 immunohistochemistry (Figure 4C-D). No changes were observed in striatum and cerebellum, which are known to be less affected by Aβ pathology (Figure S7). Quantification of 6E10 immunofluorescence revealed a significant increase in amyloid plaques number from 6 m in cingulate cortex (+533 ± 47% at 12 m and +813 ± 43% at 18 m vs 6 m p < 0.001; +44 ± 28% at 18 m vs 12 m p = 0.039), hippocampus (+364 ± 26% at 12 m and +446 ± 27% at 18 m vs 6 m p < 0.001), and later in thalamus (+321 ± 16% and +109 ± 35% at 18 m vs 6 m and 12 m, p < 0.001 and p = 0.003 respectively) (Figure 4C). Notably, the number of plaques in the thalamus was about 10 times lower than in cortex and hippocampus at all ages (Figure 3C). In addition to progressive amyloid deposition, increased plaque size (µm^2^) was also seen with age in cingulate cortex (+68 ± 16% at 12 m and +49 ± 20% at 18 m vs 6 m, p < 0.001 and p = 0.002 respectively), but not hippocampus or thalamus (Figure 4D).

### (S)-[^18^F]THK5117 PET and autoradiography detect increased Tau pathology, immunohistochemistry reveals that it is localized around amyloid plaques in TgF344-AD rats

**Figure 5:**
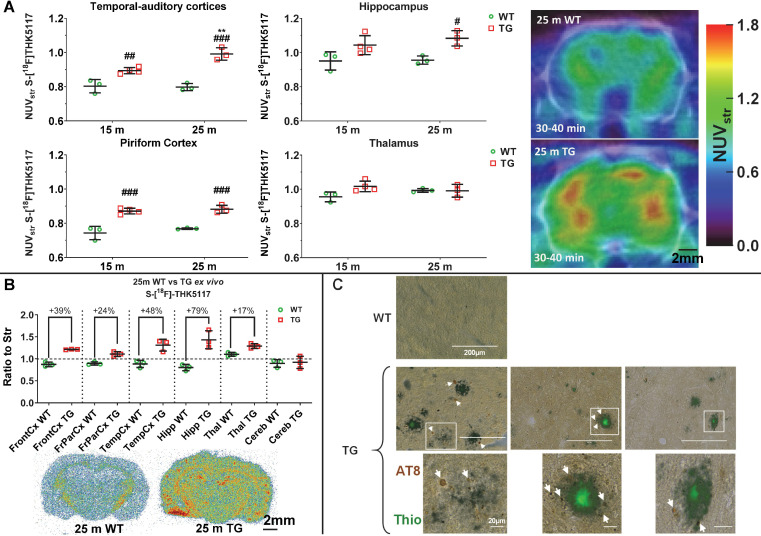
Tauopathy in the TgF344-AD rats as detected by *in vivo* by PET imaging (**A**), autoradiography (**B**) and immunohistochemistry (**C**). (**A**) PET data revealed an increase in [^18^F]THK5117 uptake in TG rats, mostly in hippocampal and cortical areas. (**B**) *Ex vivo* autoradiography revealed greater differences in Tau signal than PET in most cortical areas, the hippocampus, and a modest but significant increase in the thalamus. (**C**) Immunohistochemistry using AT8 anti-Tau antibody in 18 month (m) old TG rats revealed that Tau deposition (arrows) occurred only around amyloid plaques. PET and autoradiography data are expressed as mean ± SD. * and # indicate significant difference between 15 and 25m old animals and between WT and TG, respectively. * or # indicates p < 0.05, **/## p < 0.01 and ***/### p < 0.001. PET data were analysed using a 2-way ANOVA (genotype and age) and a Sidak post-hoc test. Autoradiography data were analysed using a Welch's *t*-test. (**C**) Scale bars represent 200 µm (top row) and 20 µm (bottom row).

(S)-[^18^F]THK5117 PET was performed to detect Tau deposition at 15 m and 25 m (Figure 5A). Longitudinal analysis revealed a significant effect of genotype and/or age on tracer uptake in temporal-auditory cortex (genotype p < 0.001, age p = 0.020, age × genotype interaction p = 0.012), frontal cortex (genotype p = 0.012, age p = 0.001), piriform cortex (genotype p < 0.001) and hippocampus (genotype p = 0.003), resulting in increased signal in TG vs WT rats in these regions (temporal-auditory cortex 15m: +11 ± 3%, p = 0.006, 25 m: +24 ± 3%, p < 0.001; hippocampus 25 m: +13 ± 3%, p = 0.019; piriform cortex 15 m: 17 ± 4%, p < 0.001, 25 m: 15 ± 2%, p < 0.001, Fig, 5A; frontal cortex, 15 m: +9 ± 2%, p = 0.003 Figure S8A). Increased uptake was observed with age only in cingulate cortices (p = 0.016) and frontoparietal cortex (p = 0.009). No significant difference was observed in the thalamus (Figure 5A), pons, cerebellum or hypothalamus via PET.

High resolution in *ex vivo* autoradiography of the animals previously scanned with (S)-[^18^F]THK5117 at 25m confirmed increased signal in TG rats in cortical areas: frontal:+39 ± 3%, p = 0.007; frontoparietal: +24 ± 5%, p = 0.008; temporal: +48 ± 10%, p = 0.014) and hippocampal (+79 ± 12%, p = 0.023) regions (Figure 5B). A moderate increase was also observed in the thalamus (+17 ± 4%, p = 0.012); however, in line with the *in vivo* PET results, no significant difference was detected in the cerebellum (Figure 5B).

As gold-standard experiments to confirm (S)-[^18^F]THK5117 PET and autoradiography experiments, we performed immunohistochemistry using phospho-Tau AT8 (Figure 5C), CP13 and PHF-1 (Figure S8B-C) anti-Tau antibodies and confirmed the presence of endogenous Tau hyperphosphorylated in various positions (AT8: Ser202+Thr205; CP13: Ser202; PHF-1: Ser396+Ser404) in hippocampus of TG but not WT rats at 18 m. Moreover, Tau pathology appeared to be localized around Thioflavine-S positive amyloid plaques in this model. Western blots of hippocampus and whole-cortex homogenates were not sensitive enough to detect a significant increase in Tau phosphorylation in TG rats (Figure S9); this is consistent with the phosphorylated Tau detected being restricted to the periphery of Aβ plaques by immunohistochemistry (Figure 5C, Figure S8) which will be diluted when working on homogenate of whole brain structures such as hippocampus or cortex.

### Neuronal loss in TgF344-AD rats is limited to regions occupied by Aβ plaques

**Figure 6:**
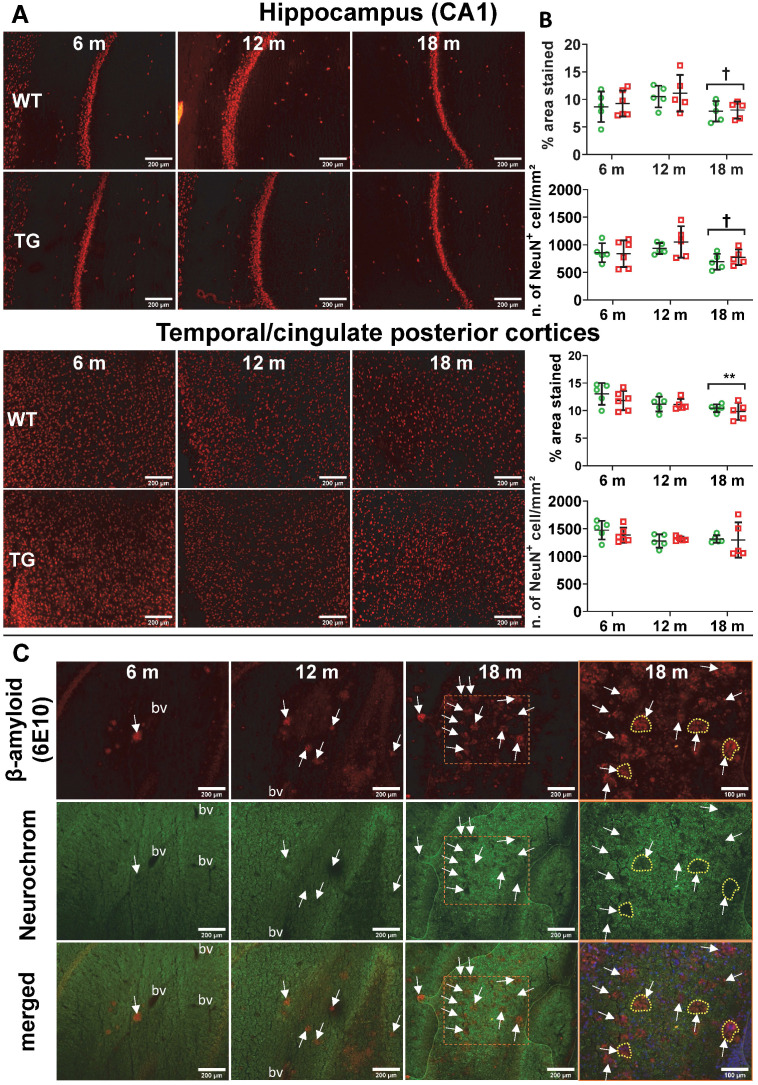
(**A**) Representative micrographs of NeuN staining in WT and TG rats at 6, 12 and 18 months (m) of age in the hippocampus CA1 (top panel) and temporal-cingulate posterior cortices (bottom panel) and (**B**) their respective quantification expressed as percentage stained area and number of NeuN positive cells/mm². There was no significant difference in NeuN staining between WT and TG and only a significant decrease due to age. Data are expressed as mean ± SD. * and ✝ indicate significant differences vs the 6 and 12 m old animals respectively. * or ✝ indicates p < 0.05, **/…… p < 0.01. Data were analysed using 2-way ANOVA (age as repeated factor and genotype) and a Sidak post-hoc test. (**C**) Representative micrographs of immunohistochemistry for pan-neuronal Neurochrom and β-amyloid in the hippocampus of TG rats at 6, 12 and 18 months (m) of age highlighting a clear loss of neuronal staining where β-amyloid plaques are present. Normal Neurochrom staining is characterised by a homogenous staining of the grey matter (bv = space occupied by blood vessels, negative for Neurochrom). Last panel on the right shows higher magnification of dotted line box of 18 m group with DAPI stain for the merged images. Yellow dotted lines highlight the loss of Neurochrom staining at the location of Aβ plaques. (**A** & **C**) Scale bars represent 200 µm.

Despite extensive attempts to detect neuronal loss using NeuN immunohistochemistry by fluorescence or chromogenic methods, we only detected age-related neuronal loss in hippocampus (CA1), cingulate posterior and temporal cortices (Figure 6A-B, Figure S10) and thalamus (Figure S10A). We found no significant effect of genotype. However, we noticed an increase in autofluorescence in the cell body of the neurons with age, it is therefore possible that this transient increase in the number of NeuN+ cells might be a false positive, this motivated the use of the chromogenic method to avoid this issue for further investigations.

The chromogenic method revealed only a non-significant trend of decrease in neuronal count in CA3 in TG at 18 m (-14% ± 12, p = 0.298) (Figure S11). Neutral red Nissl labelling of dentate gyrus, CA1 and CA3 produced similar results (data not shown).

Using the pan-neuronal marker Neurochrom, we were however able to observe clear loss of neuronal staining in the space occupied by or even surrounding Aβ plaques (Figure 6C, Figure S10B), demonstrating a direct but very localised impact of Aβ plaques on neurons. Normal Neurochrom staining is characterised by a homogenous staining of the grey matter in which blood vessels (bv in Figure 6C) and lack of staining due to plaques appear darker (white arrows in Figure 6C).

### Absence of significant cognitive decline in TG rats may be masked by reduced locomotor activity in the Fischer strain

**Figure 7:**
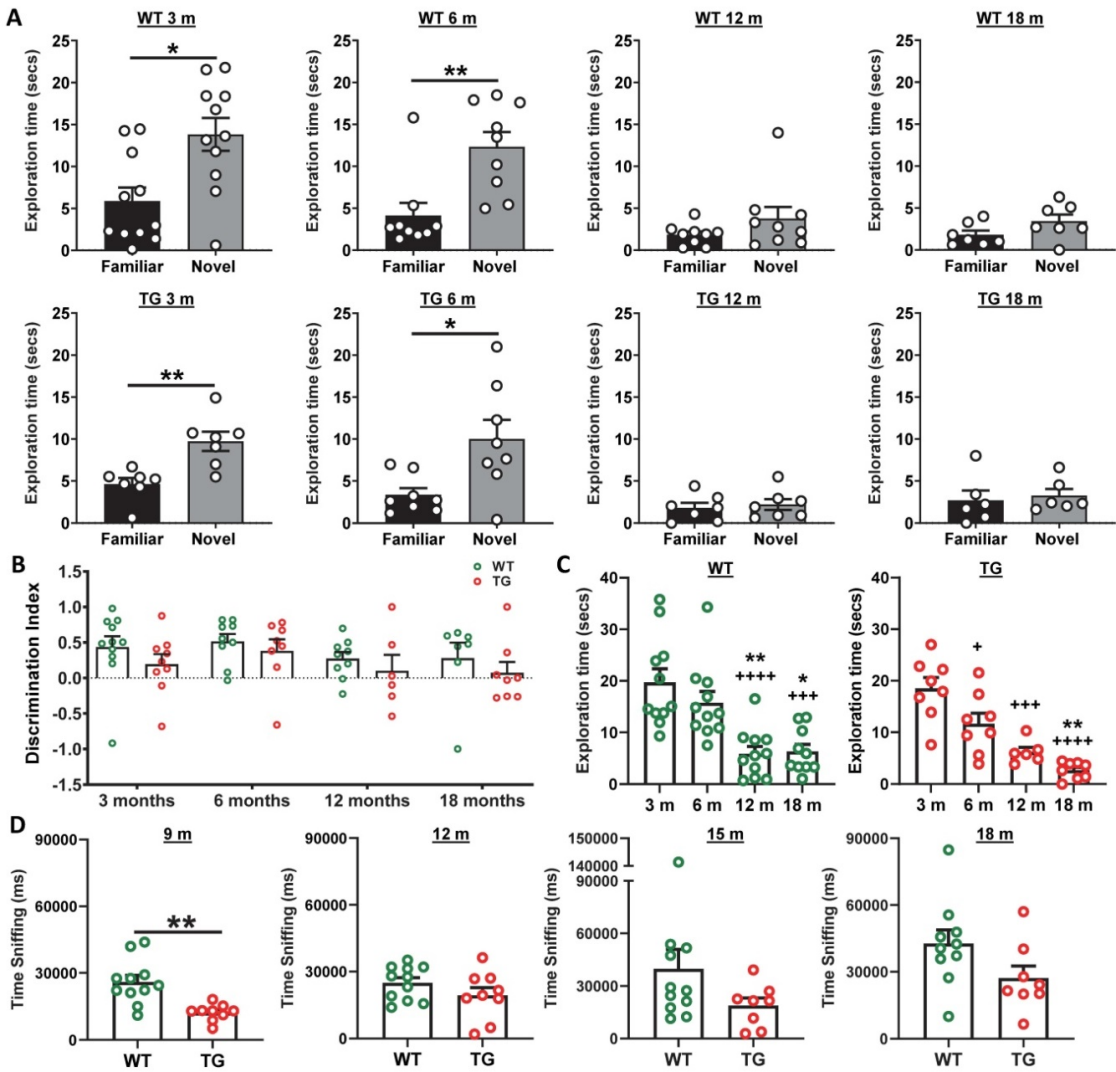
(A) Wildtype (WT) and transgenic (TG) rats displayed increased exploration of the novel object in the NOR retention phase at 3 and 6 months (m) but were unable to discriminate objects at 12 and 18 m of age (*t*-tests). Analysis of discrimination index (DI) over time did not reveal any significant differences between groups (genotype p = 0.093, age p = 0.283, 2-way ANOVA mixed model repeated measure) (B). Total exploration times in the retention phase of the NOR test revealed significantly reduced active exploration of both WT and TG rats with age (1-way ANOVA) (C). TG rats spent decreased time sniffing con-specific animals than WT rats in the social interaction test, with a significant reduction observed at 9 m (t-tests). Data are expressed as mean ± SEM*.* + indicates significant difference vs. 3 m and * indicates significant difference vs. 6 m. +/* p < 0.05, **p < 0.01, +++/*** p < 0.001 and ++++/**** p < 0.0001.

Longitudinal NOR revealed a significantly longer exploration of the novel over the familiar object in the retention phase of the task in both WT and TG animals at both 3 and 6 m of age (Figure 7A), however neither WT nor TG animals demonstrated any preference for the novel object at 12 or 18 m of age (Figure 7A). Analysis of discrimination index (DI) over time did not reveal any significant differences between groups (genotype p = 0.093, age p = 0.283, interaction age × genotype p = 0.986, Figure 7B). Total exploration times in the retention phase of the NOR test was significantly reduced in both WT (12 vs 3 m: -14 ± 62% p < 0.0001; 12 vs 6 m: -63 ± 63% p = 0.006; 18 vs 3 m: -68 ± 56 % p = 0.0002; 18 vs. 6 m: - 60 ± 57% p = 0.0117) and TG rats (6 vs 3 m: -37 ± 41% p = 0.0267; 12 vs 3 m: -67 ± 34 % p = 0.0002; 18 vs 3 m: -86 ± 47% p < 0.0001; 18 vs. 6 m: -77 ± 56 % p = 0.026) with age (Figure 7C). Similar results were found for total exploration time in the acquisition phase (Figure S12B). No significant differences were identified between the exploration of the left and right objects in the acquisition phase with either group at any age indicating no side bias (Figure S12C). In general, this reduced locomotor activity limits the interaction with objects in the NOR and may be masking any cognitive deficits between genotypes.

Following our own observations in the NOR at 3 and 6 m, we decided to investigate social interaction at 9, 12, 15 and 18 m by investigating sniffing behaviour of a conspecific WT animal, as well as quantifying the time of exploration of a central object. A reduction was observed in sniffing behaviour at 9m (Figure 7D) with TG rats spending less time sniffing and interacting with conspecific animals compared to WT (p = 0.001). However, there was no effect at other time points. No significant differences were seen in exploration of the central object between WT and TG rats at any time-point (9m p = 0.087; 12 m p = 0.905; 15m p = 0.498; 18 m p = 0.203) (Figure S12D).

### AD-like pathology and normal aging affect regional brain metabolite profiles assessed by MRS

**Figure 8:**
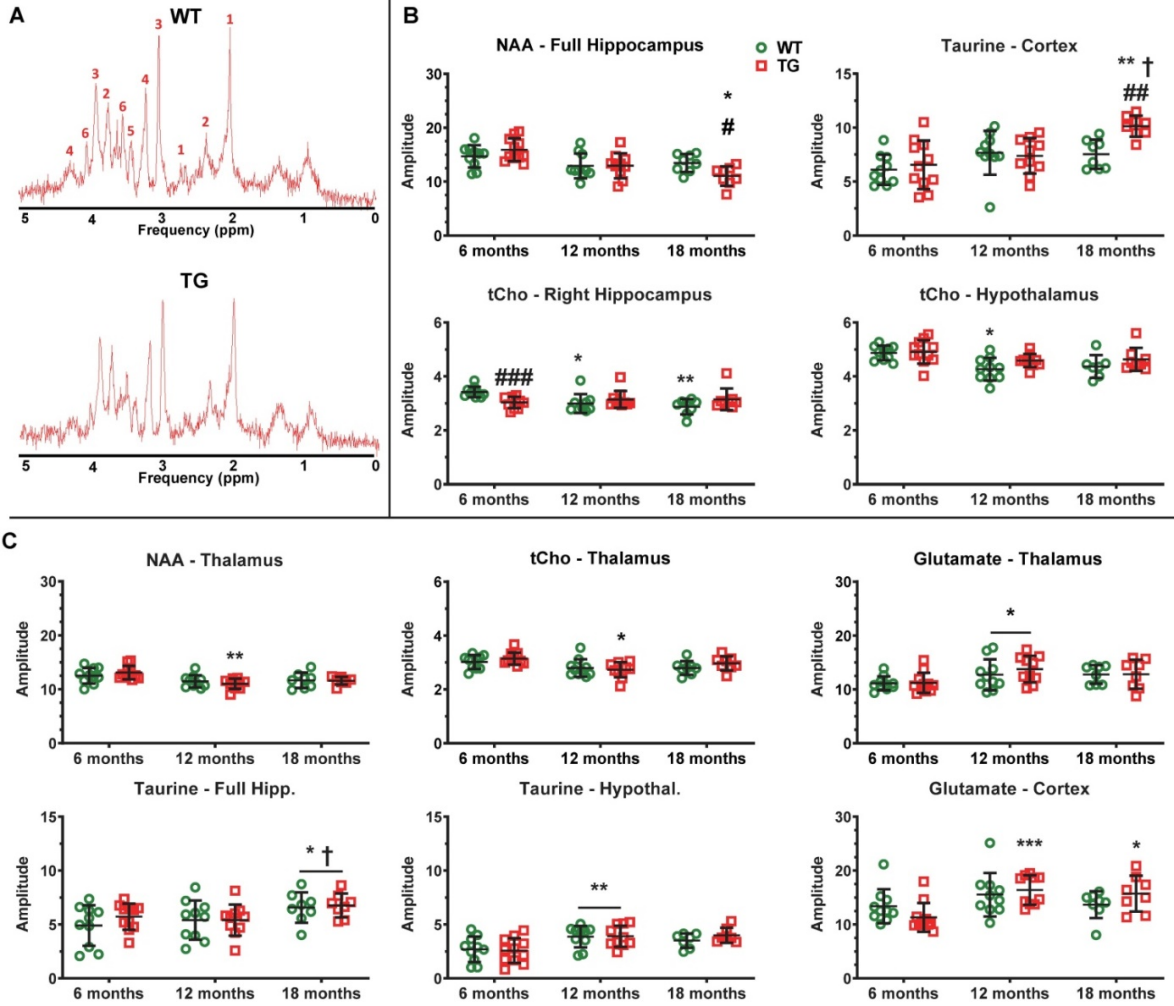
** AD-like pathology and normal aging affect regional brain metabolite profiles. (A)** Example MRS spectra obtained from the hippocampus of Wildtype (WT) and transgenic (TG) rats at 6 months (m) of age. The multiple peaks of metabolites of interest are labelled as: 1: N-Acetyl-aspartate, 2: Glutamate, 3: total Creatine (creatine + phosphocreatine), 4: tCholine, 5: Taurine, 6: *myo*-inositol. **(B)** Metabolites affected by age and genotype/age × genotype interaction. **(C)** Metabolites affected by age alone. * and ✝ indicate significant differences vs the 6and 12 m old animals respectively and # indicates significant difference between WT and TG. *, ✝ or # indicates p < 0.05, **/……/## p < 0.01 and ***/………/### p < 0.001. The concentration of metabolites presented are expressed in institutional units which relate to mMol/kg tissue wet weight, assuming a water content of 0.78 mL/g in rodent brain. MRS data were analysed using a mixed model (age as repeated factor and genotype) and a Sidak post-hoc test. Results are shown as mean ± SD. Note: brain region names in figure are written differently for some panels e.g. hipp. & hippocampus, Hypothal. & hypothalamus.

MRS was performed in WT and TG rats at 6, 12 and 18 m to assess metabolite profiles in the hippocampus (bilateral and right side), thalamus, hypothalamus and cortex (example spectra in Figure 8A). An effect of age (p < 0.001) and an age × genotype interaction (p = 0.037) were observed with N-acetyl-aspartate (NAA) in the bilateral hippocampus, resulting in significantly reduced NAA levels in 18 m TG vs age-matched WT (-18 ± 14%, p = 0.042) and vs 6 m TG rats (-31 ± 15%, p = 0.017) (Figure 8B). We did not observe alterations in NAA in other regions displaying high levels of Aβ plaques in this rat model such as the cortical voxel.

The total of choline-containing compounds (tCho) in hypothalamus was affected at both age (p < 0.001) and genotype (p = 0.047), with a modest but significant reduction in WT rats at 12 m only (vs 6 m: -12 ± 6%, p = 0.017). Similarly, tCho levels in right hippocampus were affected by age and genotype (interaction p = 0.002), with reduced tCho levels seen in TG vs WT rats at 6 m (-11%, p < 0.001) and with age only in WT animals (12 m vs 6 m: -13 ± 9%, p = 0.040; 18 m vs 6 m: -16 ± 8%, p = 0.006, Figure 8B). In contrast, cortical Taurine levels increased in the cortex (age p < 0.001, interaction p = 0.047 and a trend for genotype p = 0.058), leading to significant increases with age in TG rats (18 m vs 6 m: +55 ± 22%, p = 0.007; 18 m vs. 12 m: +37 ± 16%, p = 0.012) and in TG when compared to WT rats at 18 m (+35 ± 14%, p = 0.002).

Additionally, some metabolites were affected by age alone in the thalamus (NAA p < 0.001; tCho p = 0.003; glutamate p = 0.012), cortex (glutamate p < 0.001), full hippocampus (Taurine p = 0.031) and hypothalamus (Taurine p < 0.001). However, some changes were driven by the TG data (Thalamus: 12 vs. 6 m: NAA -16% p = 0.006; tCho -13 ± 9% p = 0.032; Cortex 12 m and 18 m vs 6 m: Glutamate +45 ± 20% p < 0.001 and +39 ± 22% p = 0.019, respectively) (Figure 8C). *Myo*-inositol levels were not significantly altered at any time-point.

## Discussion

Through the use of a multi-modal approach carried out independently in various labs, we demonstrate here that a comprehensive range of parameters, from cognition to brain pathological features, are altered in the TgF344-AD rat model. We report for the first time, longitudinally and *in vivo*, alterations of neuroinflammation, the α7 nicotinic receptor, amyloid burden, Tau pathology and brain metabolites using PET and MRS techniques in the TgF344-AD model of AD. It must however be noted that most *ex vivo* observations obtained here are similar to those seen in mouse models of AD. Hence the AD rat presents a model that is similar in most aspects to the transgenic mouse models but with the undoubted advantage of having a larger brain that allows application of a wide range of *in vivo* imaging techniques which are far less applicable to or less sensitive in mice. It must be noted however that the larger rat brain does not completely circumvent potential limitations of *in vivo* imaging such as partial volume effect, which may hamper quantification of PET signal in all species including humans especially when measuring small brain structures and/or subtle changes. Ultimately, *in vivo* imaging presents the advantages of being non-invasive, allowing longitudinal studies to explore various molecular targets in a same animal and is fully translational while *ex vivo* read-outs are the complementary, more sensitive, absolute gold standards in term of pathophysiology.

Our findings support the use of imaging in such a rat model to monitor disease progression *in vivo* and investigate new therapies more sensitively than can be achieved with behavioural tests, which are harder to measure and may appear only at later stages. The changes we have observed in the TG rat model are also in good agreement with clinical observations, further supporting the use of this model to investigate AD.

### Neuroinflammation

Evidence suggests that neuroinflammation has an integral role to play in AD development with reports of increased neuroinflammation early in clinical AD and MCI as well as in pre-clinical models 33, 49, 50. Cohen *et al.* 12 detected a moderate increase in neuroinflammation using immunohistochemistry as early as 6 m in the TgF344-AD rat model. Hence, we wanted to investigate if we could detect these *in vivo* using [^18^F]DPA-714 PET. Here, we observed increased [^18^F]DPA-714 uptake in the hippocampus (12 m) and cortical areas and thalamus (18 m) of TG rats compared to WT rats. These results are in agreement with numerous previous TSPO PET study in mouse models of AD 26, 29, 51-56 and also the majority of clinical studies 33, 57-59 which reported similar (+10-30%) significant increase in TSPO tracer uptake mostly in regions affected by AD pathology (e.g. hippocampus, frontal and cingulate cortices). Interestingly, increased [^18^F]DPA-714 binding was also seen with age in WT animals, suggesting increased inflammation occurs with normal aging. This is in line with findings of increased neuroinflammation with normal aging 60-62 as notably assessed by TSPO PET in in WT animals (+4-20%) 63, 64 as well as healthy subjects (+4.7~10% per decade) 65, 66. Similarly, *ex vivo* analysis by immunohistochemistry demonstrated an early (6 m) modest presence of activated microglia/macrophages, increasing sharply by 12 m and even further at 18 m in the hippocampus and cortical areas in TG. Increases in [^18^F]DPA-714 uptake was matched by increase in CD11b staining in most ROIs, except for the hippocampus at 12 m in which there was a trend to increase in CD11b (p = 0.098) and the temporal cortex in which the [^18^F]DPA-714 uptake was not significantly altered at 12 m. Various reasons such as difference in sensitivity of the methods and/or statistical variability are likely to have caused such discrepancies. Interestingly, and in agreement with our PET results, microglial activation was also found to be elevated in thalamus at 18 m, although more modestly than in the hippocampus and cortices, and in relation with the much lower amyloidosis (× 10 less in thalamus than hippocampus/cortices). Astrogliosis was also increased in TG vs WT but conversely to microgliosis, astrogliosis was elevated in TG at all ages and as early as 6 m but tended to decrease with age in both TG and WT.

These results are altogether in agreement with our PET results and numerous previous reports showing increasing microglial activation with AD burden and age in various rodent models including the TgF344-AD rats 12, 26, 29, 63, 67-73. Similarly, the decrease in astrocyte staining with age and/or progression of AD pathology observed here is consistent with previous reports that have demonstrated a complex regulation of astrocytes with age and disease with astrogliosis in disease combined with a decrease in astrocyte numbers and function with age 74-78.

### Amyloid pathology

Here we report the first longitudinal investigation of amyloid-PET in the TgF344-AD model. Using *in vivo* [^18^F]Florbetaben PET, we detected a significant cortical and hippocampal amyloid deposition in TG compared to WT rats at 18 m (+12-15%) of age, similar to those detected in mice (+12~25%) at 18-20m of age 29, 63, 79, 80 but lower than those reported in clinical studies (+25~100%) 57, 81, 82. In support of this, immunohistochemical analysis revealed significant progressive amyloid deposition in cortical and hippocampal regions of TG rats starting from a sparse but significant amyloid deposition at 6 m progressing towards a heavy amyloid load at 12 m and 18 m of age. Our immunohistochemical results are in agreement with the initial report in this model 12. However, amyloid PET was not able to detect significant increases at 12 m *in vivo*, likely due to *i)* the limitations in resolution of small animal PET imaging, *ii)* the poor signal to noise ratio of amyloid tracers which are notoriously lipophilic and *iii)* 12 m being a relatively young age for the rats. In this regard, and compared to clinical amyloid PET scans, it must be considered that 6 m and 12 m are relatively young ages in rats which have a life-expectancy that can reach about 30m depending on the strain 83, although the TgF344-AD rats have been aged only up to 25m here (Turku) and up to 26 m in the literature 12; so one may consider that these rats were imaged proportionally at younger than AD or MCI patients would.

### Tau pathology

The TgF344-AD rat was reported by Cohen *et al.* 12 to be the first rodent model to exhibit spontaneous NFT-like hyperphosphorylated Tau accumulation similar to that seen clinically. Here we use (S)-[^18^F]THK5117 to investigate longitudinally the Tau burden *in vivo*. We observed significant increase in (S)-[^18^F]THK5117 uptake in cortical and hippocampal regions TG rats when compared to age-matched WT rats mostly at 25m of age. *Ex vivo* autoradiography supported these findings and revealed markedly elevated binding in regions known to be affected in AD including the frontal cortex, hippocampus and thalamus. These results are somehow in contradiction with the Tau biochemistry analysis performed by Cohen *et al.* 12 which demonstrated increased Tau level earlier, at 6 and 16 m, but no further increase at 26 m, however this is most likely due to the methodological approaches being very different (*in vivo* or *ex vivo* isotopic methods vs Western blots). However, our immunohistochemical analysis identified Tau hyperphosphorylation in the hippocampus at 18 m in agreement with previous studies 12, 28. Our immunohistochemical analysis also revealed that hyperphosphorylated Tau was found only in dystrophic neurites around the amyloid plaques in the TG rats. This is in agreement with the potential seeding of Tau pathology by Aβ 84 and observations by Cohen *et al.* 12 and also Morrone *et al.* 19 who reported that 80-85% of PHF1+ neurons were located near Aβ plaques. This observation is however in contrast to Tau burden reported also in non-plaque regions of the cortex and hippocampus 12, 28. Those differences between studies may potentially be related to differences in the methods used and/or ages studied here and in other studies. The differences between the results of *in vivo* Tau PET and immunohistochemistry are likely to be explained by the lower sensitivity of PET and by the previously reported off-target binding of (S)-[^18^F]THK5117 85, 86. Such off-target binding, notably to monoamine oxidases, has been a major problem to most first-generation Tau tracers 87, 88. The results presented here clearly confirms that off-target binding of Tau tracers may hamper detection of Tauopathy, and although second generation of Tau tracers have shown negligible monoamine oxidase binding 88, 89, binding to other molecules such as neuromelanin and melanin still present a challenge to development of a Tau-specific PET tracer 87, 90, 91. It is generally accepted that hyperphosphorylated Tau or NFT are absent or extremely difficult to detect in the mouse models with identical transgenes, although in some models, modest levels of hyperphosphorylated Tau have been reported 92. Some PET studies using other tracers have successfully imaged Tau accumulation in transgenic Tau mouse model, and it is possible that using one of the new Tau PET tracers may show *in vivo* tau accumulation in the TgF344-AD rats at earlier time-points. Conversely, the levels of Tau deposition in this model have now been consistently reported by us and others 12, 19, 28, suggesting that this rat model reproduces this essential feature of AD. Moreover, the 3R to 4R Tau ratio in rats is similar to humans, but different to mice. Similarly to this model, the APP × PS1 rat model generated by Flood *et al*. 93 displayed dense fibrillar Aβ plaques with phosphorylated Tau in close proximity. Conversely, in the McGill-R-Thy1-APP model generated by Leon *et al.* 94, no neuronal loss or NFT-like Tau pathology were reported despite displaying amyloid pathology, cognitive deficits, and increased neuroinflammation.

### Neurodegeneration

Here, and despite using 2 different methods of immunostaining for NeuN, we were not able to detect a significant decrease in numbers of neurons between WT and TG, contrary to the initial report on the TgF344-AD rats 12. We however detected a significant decrease in NeuN percentage-stained area with age in both WT and TG in hippocampus, posterior cingulate/temporal cortices and thalamus at 18 m of age, which may have contributed to the lack of differences between WT and TG at this age. Since the publication of the initial characterisation of the TgF344-AD strain, reports regarding neuronal loss/neurodegeneration have produced mixed results. Leplus *et al.* 95 detected loss of neurons in the *gyrus dentatus* and the cortex but not in CA1 using 18 m old animals, whereas Voorhees* et al.* 96 reported neuronal loss in the hippocampus and not in the cortex at 24m of age. Voorhees *et al.* 96 noted that NeuN staining could produce false negative following the phagocytosis of neuronal debris by microglia and had to rely on cresyl-violet staining. Our results are however in line with those independently produced by our collaborators Anckaerts *et al.* 22 who did not detect any significant loss of neurons by NeuN immunostaining in female TgF344-AD at 10 and 20m of age. We however confirmed using Neurochrom staining that amyloid plaques create a void of viable neurons around them (Figure 6C and Figure S9B). This, in itself, is likely to disturb neuronal functions and connectivity. This hypothesis of very localised neuronal disruption fits well with our MRS findings showing decreased levels of NAA in the hippocampus, the presence of hyperphosphorylated-Tau positive neurons around the Aβ plaques shown here and the previously reported decrease in connectivity by rsfMRI 22, 23, 97. Subtle localised neuronal alterations are also in agreement with other reports showing a decrease in glutamic acid decarboxylase positive neurons in CA1 13, and in norepinephrine transporter and dopamine β-hydroxylase positive fibres in the hippocampus and *gyrus dentatus* respectively, without gross neuronal loss in the *locus coeruleus* 28. In line with these changes in specific neurotransmission systems, we here demonstrate that there is significant age-dependent increase in α7-nAChR in the striatum and *pallidum/NBM* in WT, which does not occur in TG. It is noteworthy that our imaging study of α7-nAChR performed for the first time longitudinally in rodents is consistent with a human PET study that revealed an increased uptake of [^18^F]ASEM according to age in various brain regions including the striatum 98. This apparent adaptation to aging, not occurring in TG, leads to a significantly lower level of α7-nAChR in TG at 18 m in the *pallidum/NBM*, which, if it had been taken in isolation, may have been considered as a decrease in TG rats. One may also consider that the changes attributed here to the *pallidum/NBM* may actually reflect changes occurring more specifically in the NBM (*substantia innominata*) principal efferent cholinergic structure in the rat brain and which was included in this larger ROI because the size of the NBM alone is beyond the resolution of PET and because of its close proximity with the *globus pallidus* 99. It is particularly relevant to consider this in light of previous reports showing alterations of cholinergic receptor density in the striatum and thalamus which have been reported in AD 100-104.

We thus brought here new evidence of the involvement of the cholinergic system during aging and their dysfunction in AD, hence further supporting its interests as therapeutic target in AD. In particular, the involvement of α7-nAChR in this context may suggest this family of receptors as a potential target in AD 105-107. Overall, this strongly supports the need for longitudinal *in vivo* multimodality investigations of the cholinergic system in animal models, as well as clinical settings to fully understand the changes in both ACh and its receptors in AD. In particular, the measurement of ACh levels in the basal forebrain in animal models and should be considered in future investigations.

### Behaviour

Cognitive deficits in the TgF344-AD model have been previously reported from 6 m in the Barnes maze and at 13 m 19 and 24m using NOR 12. Here, we assessed NOR at the earlier time-points of 3 m, 6 m, 12 m and 18 m. At both 3 and 6 m, we did not observe significantly reduced performance in these rats suggesting there is no cognitive decline in TG compared to WT rats at these ages. However, we did observe reduced locomotor activity in both WT and TG rats from 12 m, which reduced their ability to perform the task. We observed deficits in social interaction at 9m of age with no changes at other time points. These results suggest that *i)* subsequent time-points between 18 m and 24m and *ii)* more refined tests, such as Morris water maze (MWM) and reversed-MWM 28 or delayed non-matching-to-sample task 23, 97, might be better suited to investigate the TgF344-AD rats. However, even with more sophisticated tests the cognitive deficits observed in TgF344-AD when compared to WT at various ages remain modest 23, 28, 97 requiring a thorough analysis of all parameters recorded 21 in order to observe significant differences. Reduced locomotor activity has previously been reported in Fischer male rats compared to other strains 46, 108 and could affect the ability of the rats to perform the NOR test. The fact that the same rats were monitored longitudinally at various age in the same environment may also explained a certain lack of motivation to perform the tests, although rats were exposed to totally new objects in the NOR tests and new conspecific animals for the social interaction test at each time-points. Altogether, our results are nevertheless in line with previous reports showing low motor activity 109, and anxious-depressive-like behaviour in the TgF344-AD 110. The previously reported anxiety of the Fischer-344 strain 45, 46, 111 coupled with reduced locomotion in both WT and TG animals experienced here suggests that back-crossing transgenic rats generated on a Fischer-344 background in another strain such as Wistar or Lister-hooded should be considered when behavioural parameters are particularly important read-outs.

### Metabolite alterations

MRS permits the non-invasive detection of biochemical changes *in vivo*, allowing potential identification of changes in regional brain metabolites prior to anatomical or disease manifestation. We here report the first MRS investigation of the TgF344-AD rats. We notably observed alterations in hippocampal NAA and tCho, and cortical Taurine levels between genotypes with age. Interestingly, decreased hippocampal NAA levels were observed in TG but not in WT rats with age. NAA has long been considered as a marker of neuronal density 112, though it may better be described as a marker of neuronal function 112-115. Therefore, decreased NAA levels are indicative of neuronal/brain dysfunction, hence our results suggest that hippocampal neuronal dysfunction occurred with age and worsened in the presence of AD-like pathology in TgF344-AD rats. This is consistent with multiple reports of decreased hippocampal NAA levels in AD and MCI patients 116-123, and in mouse models of AD 124-127 including our recent study using a mouse model 26 with the same mutations as the TgF344-AD rat 26. Similar changes in NAA were also observed in the other TG rat model of AD McGill-R-Thy1-APP at 3 m or 9m of age 128, 129.

In the hippocampus, tCho decreased with age in WT but not in TG; TG animals had however significantly lower level of tCho than WT at 6 m suggesting an early alteration of tCho in TG with no further changes with age. Decreased tCho levels have also been reported in AD patients 117 but results are inconsistent with others reporting no significant changes 120. In animal models of AD, levels of choline compounds were also decreased at 3 or 9m of age in McGill-R-Thy1-APP rats 128, 129. Conversely, Esteras *et al.* 130 and Forster *et al.* 131 found increased levels of tCho in AβPP/PS1 and TASTPM mice respectively, whereas we did not find any significant change in APP_swe_ × PS1_Δ9_ mice 26. Free choline is needed for the production of the neurotransmitter acetylcholine 132, so a decrease in tCho level may suggest reduced choline availability for acetylcholine synthesis. However, since free choline is only a minor component of the tCho peak (< 10% 133), this interpretation has to remain speculative.

*Myo*-inositol has been suggested to be a glial specific marker and that increasing levels may be indicative of microglial activation or gliosis 116. In this context, one could hypothesize an increase in *myo*-inositol in the TgF344-AD rat similarly to the transient (12 m) increase in *myo*-inositol reported in McGill-R-Thy1-APP rats 129. However, despite increased neuroinflammation and amyloid pathology demonstrated in this model by this study and previously 12, no significant differences in *myo*-inositol levels were identified at any time-point. Again, previous findings using other models produced different results, we did not observe any change in *myo*-inositol in APP_swe_ × PS1_Δ9_ mice 26 whereas Forster *et al.* 131 found a robust increase in *myo*-inositol levels in the more aggressive TASTPM mouse model. However, the notion that *myo*-inositol is a glial marker is based on a single published report by Leibfritz and Brand 134 and a link with gliosis has never been further solidified. Furthermore, recent studies have questioned the association between *myo*-inositol and neuroinflammation while a correlation with amyloid burden has been reported 116.

On the other hand, we did see increased Taurine in TG rats. Taurine is an essential amino-acid known to have numerous functions such as osmoregulatory, neurotrophic and neuroprotective roles and excess or deficiency of Taurine levels leads to several diseases 135-138. Taurine has also previously been shown to protect against excitotoxicity induced by amyloid 139, therefore the increase we observed in the cortex of TG rats at 18 m may be a compensatory protective mechanism to counter the amyloid-induced damages. In the hippocampus and hypothalamus, the increase in Taurine levels was driven by age suggesting a potential compensation to also counter aging processes. Our results are in line with increases in Taurine reported at 9 and 12 m of age in McGill-R-Thy1-APP rats 129 but have not been recapitulated in mouse models 26, 131.

Multiple regional metabolite alterations were identified with aging alone. This is in accordance with previous reports in both preclinical models 26, 129 and humans 140, 141, and highlights the need to better characterize the effects of normal aging to enable accurate measure of AD-specific alterations. Moreover, while MRS seems to be a valid tool to assess metabolite levels *in vivo*, the complexity of the analysis, differences in quantification methods (such as normalisation to water, total metabolites or creatine) as well as difficulties related to the size of the mouse brain when compared with rats and even more so between rodents and human may explain some of the discrepancies observed between studies. This highlights the need for further investigations combining *in vivo* MRS and PET with various *ex vivo* techniques such as mass-spectrometry to measure small metabolites and further understand their role in AD pathology.

## Conclusions

Altogether our results provide an extensive review of the AD-like pathology and phenotype of the TgF344-AD rats model from early to advanced stage of the disease which is important to characterise for understanding disease progression and for testing new treatments. Our results show here, for the first time, interesting characteristics in term of altered metabolites by MRS, alongside quantification of α7-nAChR, Tau, Aβ and neuroinflammation by *in vivo* PET imaging confirmed by *ex vivo* analysis. Hence, providing a full, multi-modal characterisation of this model from early age (6 and 12 m) up to a more advance stage (18 m). The overall results of this study are summarised in Table 1. This study was made possible only through extensive collaboration between many labs. We would like to note however that a true multi-centre study, implying repeating similar experiments in different institutions, would be needed to investigate variability of data collection within each technique and provide added robustness to the results. Although it must be acknowledged that such study would significantly increase the financial cost as well as the ethical cost in term of number of animals used. Such characterization is essential before further studies, which are both time-consuming and expensive, could be reasonably undertaken using this model. The data provided here support the potential of this model to investigate mechanisms of AD pathology and new therapeutics with the significant advantage of the rat larger brain, which allows more precise quantifications in* in vivo* longitudinal imaging studies, when compared to mice.

## Supplementary Material

Supplementary figures and tables.

## Figures and Tables

**Table 1:**
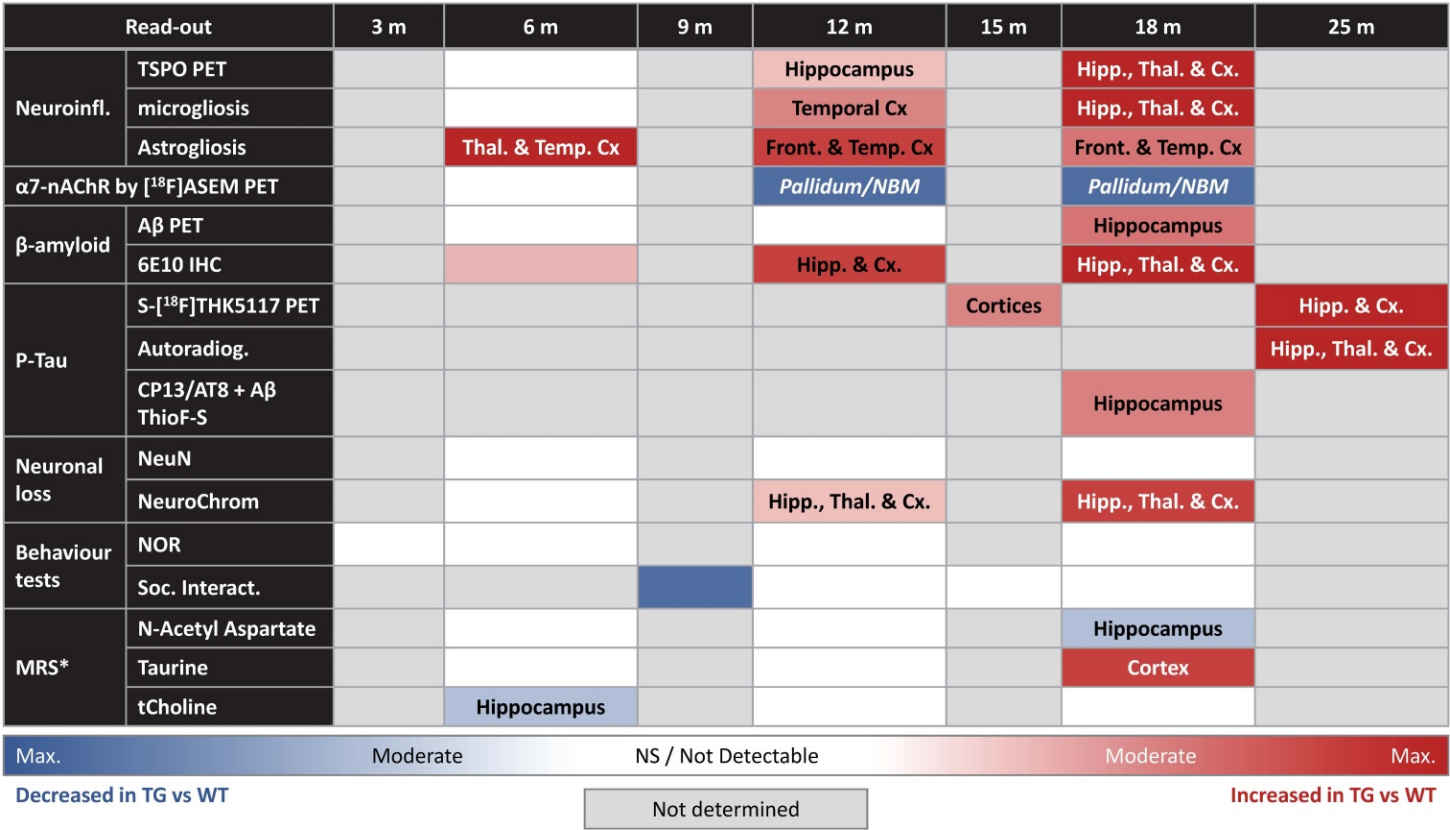
Summary of the study findings

The age in months (m) at which each parameter (in rows) was assessed is indicated in columns. Changes found at these time-points: a grey shading indicate that there was no assessment of the parameter shown in the corresponding row, a white shading indicates that the parameter was assessed but no significant change were found, a blue or red shading indicate respectively a significant decrease/lower values or increase/higher values in the TG vs age-matched WT. Brain areas where the significant changes were detected are indicated in each cell (abbreviations: α7-nAChR = α7 nicotinergic acetyl-choline receptor; Autoradiog. = autoradiography; ThioF-S = thioflavine-S; NOR = novel object recognition test; Soc. Interact. = social interaction behavioural test; Thal. = thalamus; Hipp. = Hippocampus; Temporal Cx. = temporal cortex; Front. Cx = frontal cortex; Cx. = all neorcortical areas). * For MRS, the regions of interest correspond to the voxel shown Figure S2.

## Abbreviations

AD: Alzheimer's disease
Aβ: amyloid-beta plaque
CAA: cerebral amyloid angiopathy
PET: positron emission tomography
APP: amyloid precursor protein
PS1: presenilin 1
NFTs: neurofibrillary tangle
TG: transgenic
MRS: magnetic resonance spectroscopy
α7-nAchR: α7 nicotinic acetylcholine receptor
NAA: N-acetyl-aspartate
tCho: choline-containing-compounds
WT: wild-type
