# Supplementary material for: Prodromal neuroinflammatory, cholinergic and metabolite dysfunction detected by PET and MRS in the TgF344-AD transgenic rat model of AD: a collaborative multi-modal study
Source: Theranostics. 2021 May 3;11(14):6644–67. doi: 10.7150/thno.56059 (PMC8171096; doi:10.7150/thno.56059)
Supplement: Supplementary file 1 — Supplementary figures and tables. [file thnov11p6644s1.pdf]

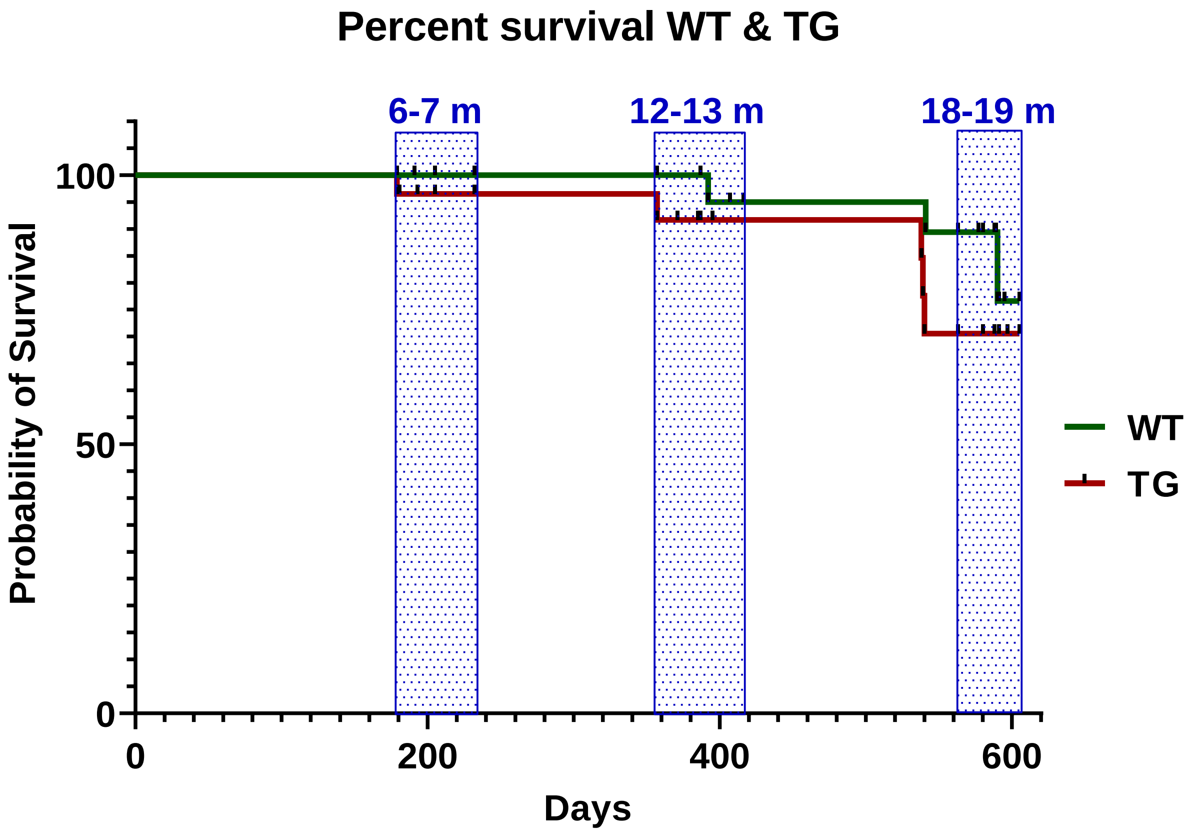

Figure S1: survival rates of WT and TG of the University of Manchester cohort. No difference in percent survival was observed between WT and TG over the course of the study (p = 0.2748, Log-rank Mantel-Cox test) (oldest animals: 605 days = 19 months 3 weeks and 6 days). The blue dotted bars represent the periods at which animals were culled for *ex vivo* analysis and therefore removed from the survival analysis.

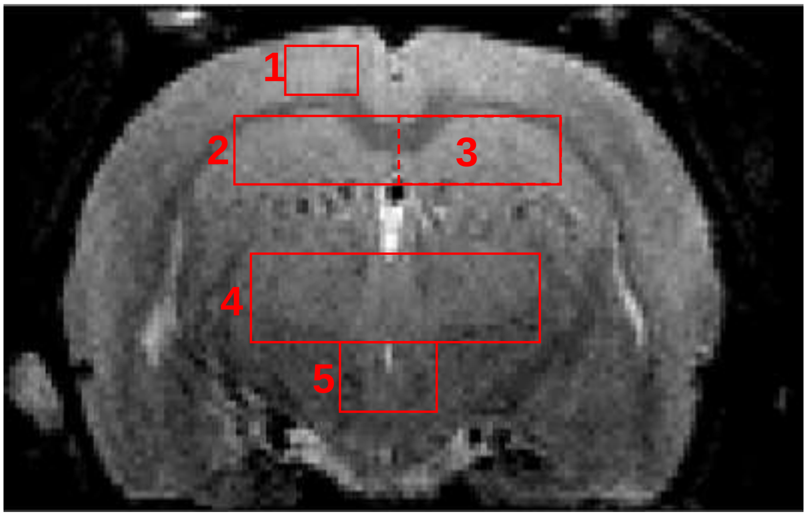

Figure S2: Rat brain MRI image indicating voxel placement for MRS acquisition in the 1: cortex, 2: full hippocampus, 3: right hippocampus (separated from full hippocampus by dashed line) 4: Thalamus and 5: Hypothalamus.

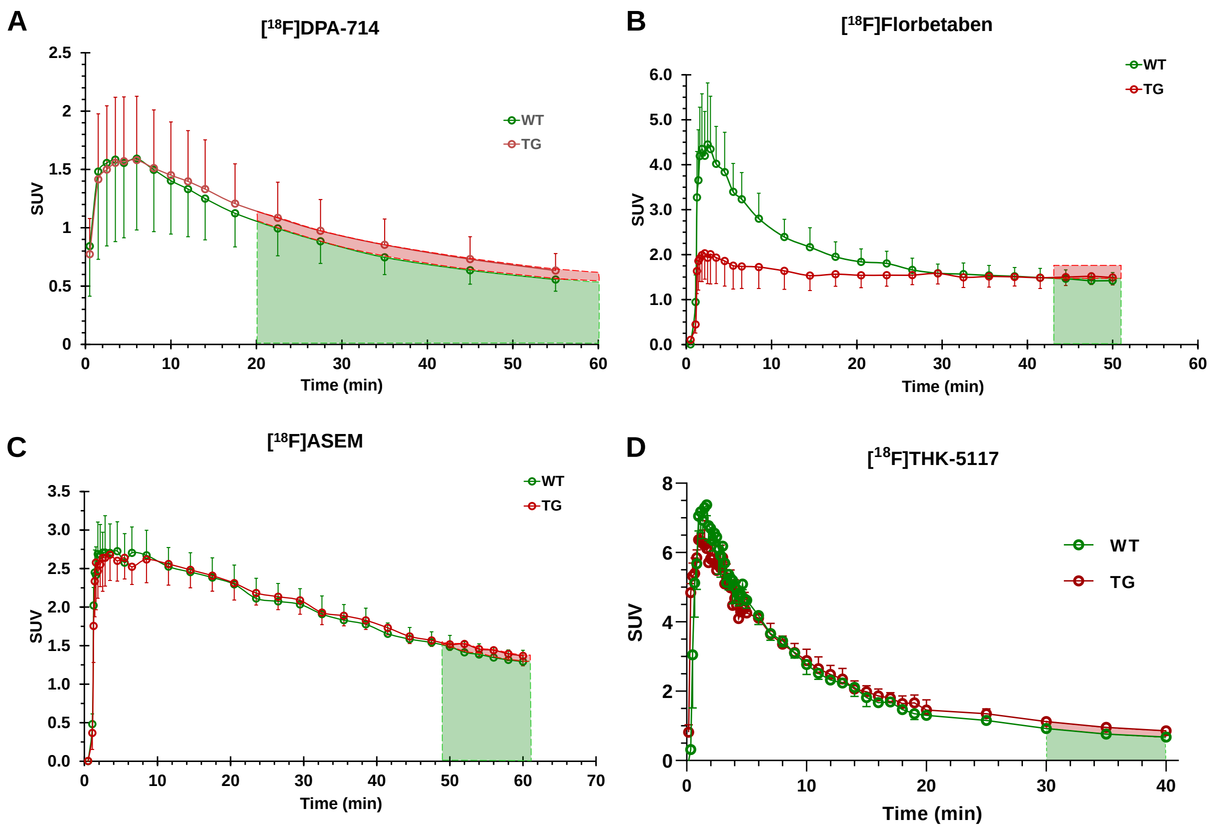

Figure S3: Time-activity curves (TAC) for [^{18}F]DPA-714 (**A**), [^{18}F]Florbetaben (**B**), [^{18}F]ASEM (**C**) and [^{18}F]THK-5117 (**D**) in the hippocampus of 18 months old WT and TG rats. The red and green shaded areas illustrate the time-frame of the scan used to calculate the average standard uptake values (SUV) and NUV for each tracer shown as individual value in Figures 1, 3, 4, 5 and S4, S6-S8 for each group. Data are shown as mean ± SD of the SUV.

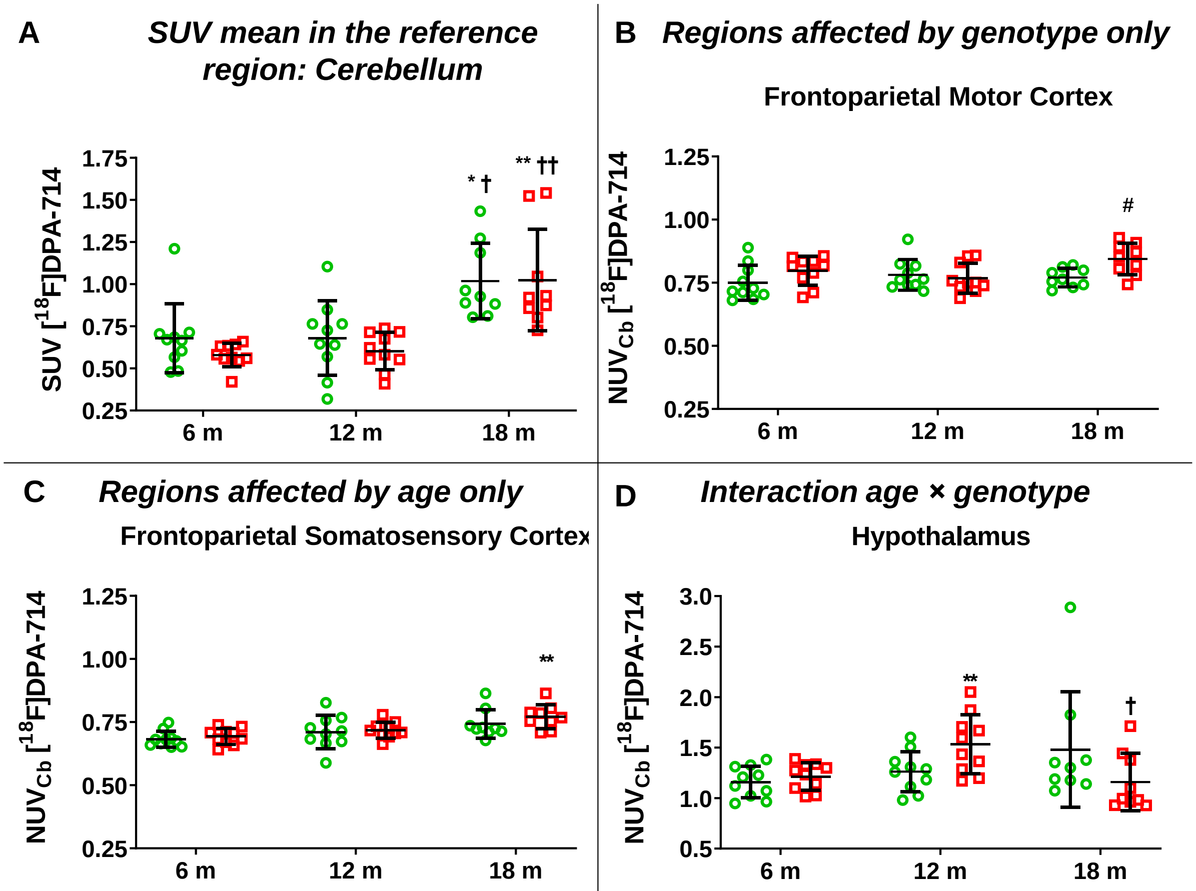

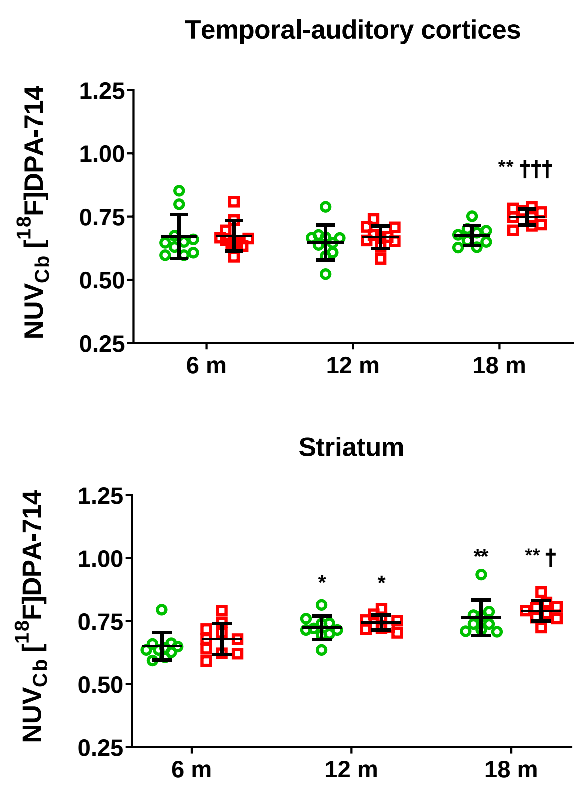

<u>Figure S4</u>: [^{18}F]DPA-714 uptake quantification in various regions of the brain in WT (green circle symbols) and TgF344-AD (red square symbols) rats at 6, 12 and 18 m. (**A**) Standard Uptake Value (SUV) of [^{18}F]DPA-714 in the cerebellum, each individual values from the cerebellum were used to normalised the other brain regions (NUV_{Cb}); only an effect of age was detected in the cerebellum in both WT and TG at 18 m. (**B**) In the frontoparietal motor, cortex only an effect of genotype was detected at 18m. (**C**) In the frontoparietal somatosensory and temporal cortices and the striatum, only an effect of age was detected. (**D**) in the hypothalamus, there was no main effect of age or genotype but an interaction age × genotype was detected. The *post-hoc* test revealed only difference between ages in TG. Data are expressed as mean ± SD. * and † indicate significant differences vs the 6 months and 12 months old animals respectively and # indicates significant difference between WT and TG. *, † or # indicates p<0.05, **/††/## p < 0.01 and ***/†††/### p < 0.001. PET data were analysed using a mixed model (age as repeated factor and genotype) and a Sidak *post-hoc* test.

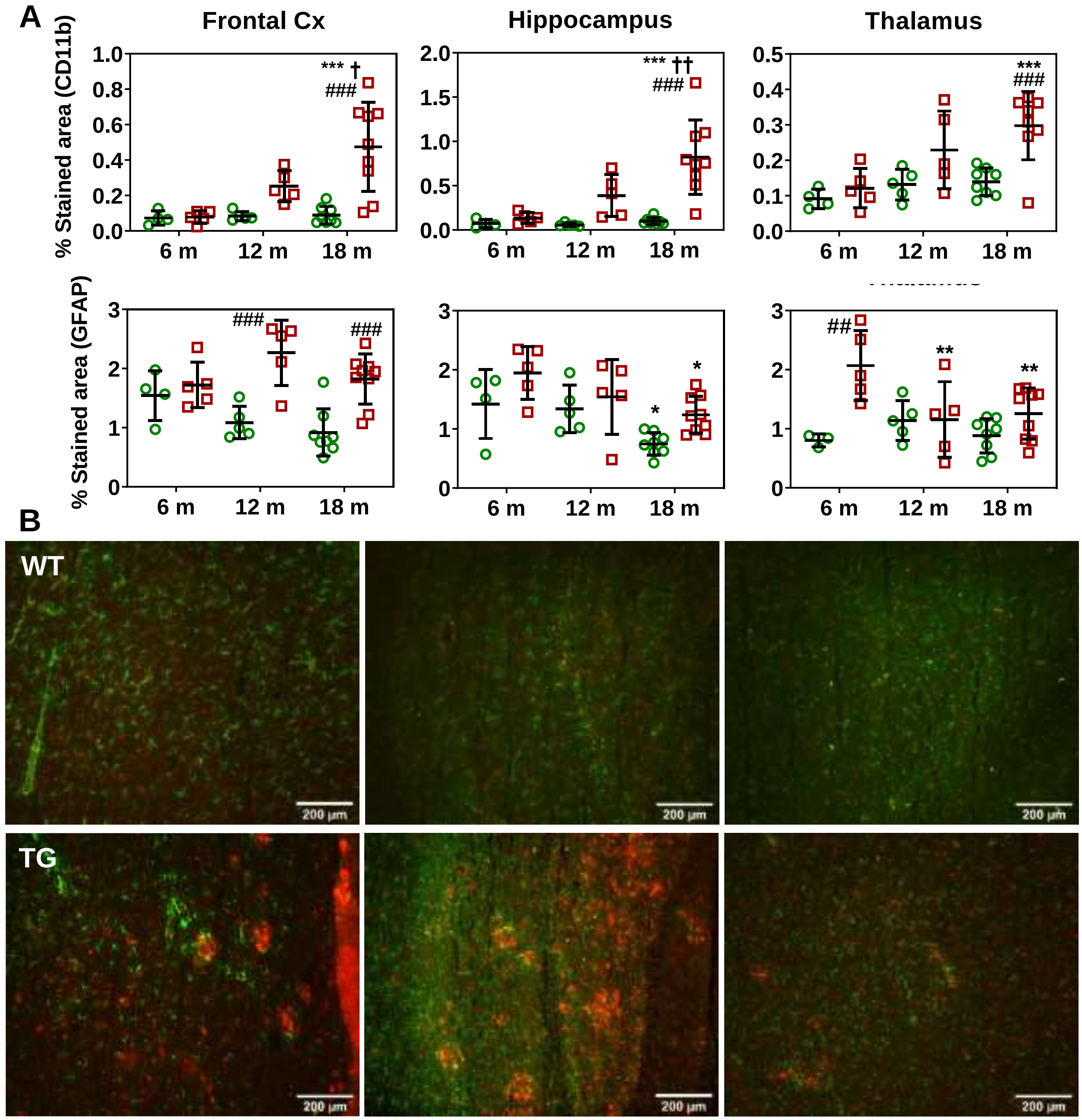

<u>Figure S5:</u> (A) Quantification of the microglia (CD11b, top row) and astrocytes (GFAP, bottom row) immunofluorescence expressed as percentage of area stained in frontal cortex, hippocampus and thalamus. * and † indicate significant differences vs the 6 months and 12 months old animals respectively and # indicates significant difference between WT and TG. Data expressed as mean ± SD. *, † or # indicates $p < 0.05$, **/††/## $p < 0.01$ and ***/†††/### $p < 0.001$. Data were analysed using 2 way ANOVA (age and genotype) and a Sidak post-hoc test. **(B)** Representative images of CD11b (red) and GFAP (green) co-staining in 18m old WT and TG rats in frontal cortex, hippocampus and thalamus (left to right).

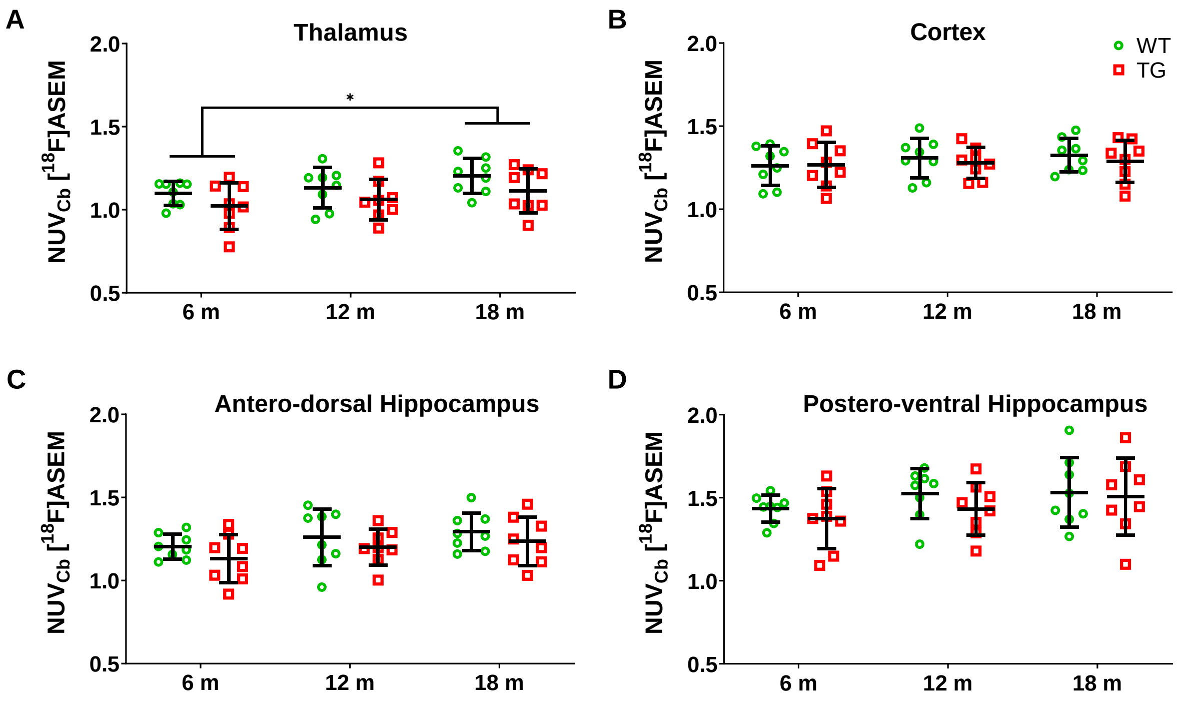

Figure S6: [^{18}F]-ASEM (α7 nicotinic receptor) uptake in the thalamus (**A**), cortex (**B**) and hippocampus (**C** & **D**) of WT (green circle symbols) and TgF344-AD (red square symbols) rats at 6, 12 and 18 months of age. Data are expressed as mean ± SD of uptake values (49-61 min) normalised to cerebellum. No significant differences were observed in these brain regions. PET data were analysed using a 2-way ANOVA (age as repeated factor and genotype) and a Sidak *post-hoc* test.

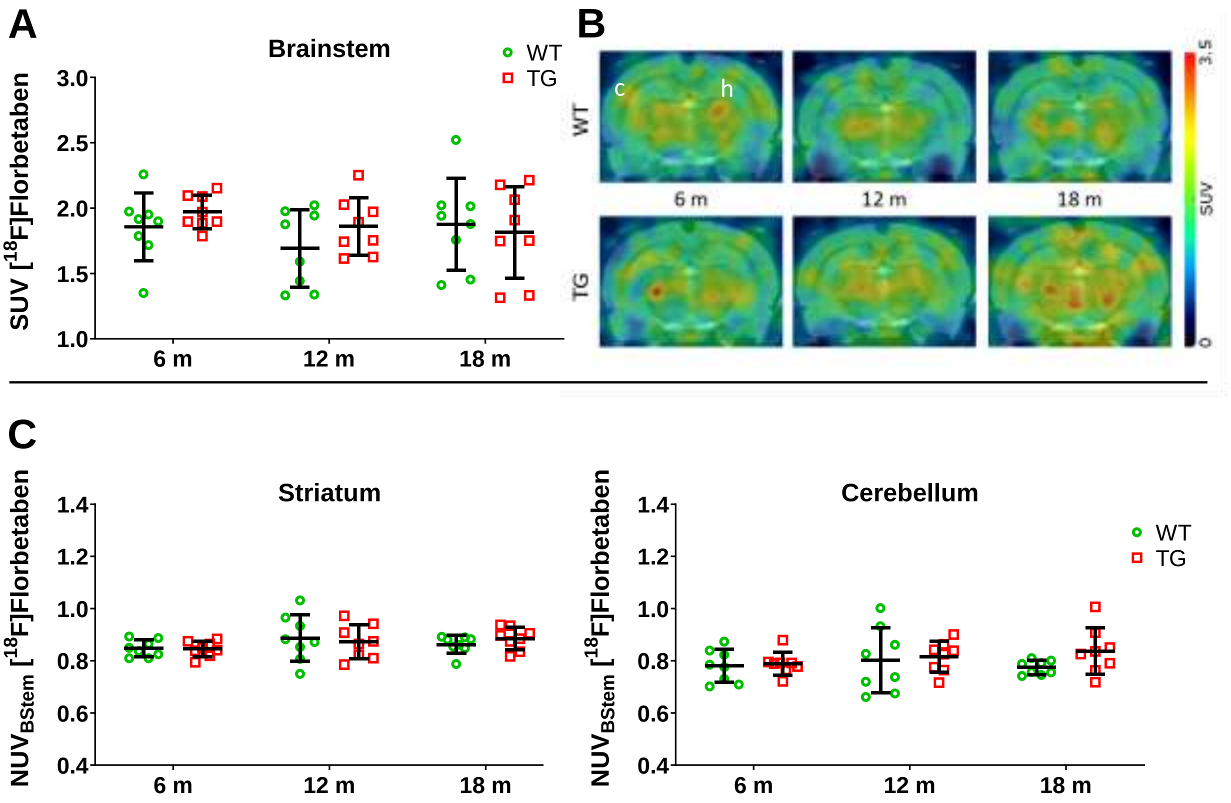

<u>Figure S7:</u> PET quantification of [^{18}F]Florbetaben uptake (PET sum images 49-61 min) in (**A**) the region of reference (brainstem) and (**B**) representative PET-MR images of [^{18}F]Florbetaben uptake (SUV). (**C**) PET quantification of [^{18}F]Florbetaben uptake in the striatum and cerebellum, 2 regions unaffected by AD-like pathology, normalised to brainstem (NUV_{BStem}) in WT (green circle) and TG (red squares) rats of at 6, 12 and 18 months of age. Data are expressed as mean ± SD. PET data were analysed using a mixed model with age as repeated factor and genotype and a Sidak post-hoc test. No significant changes were detected.

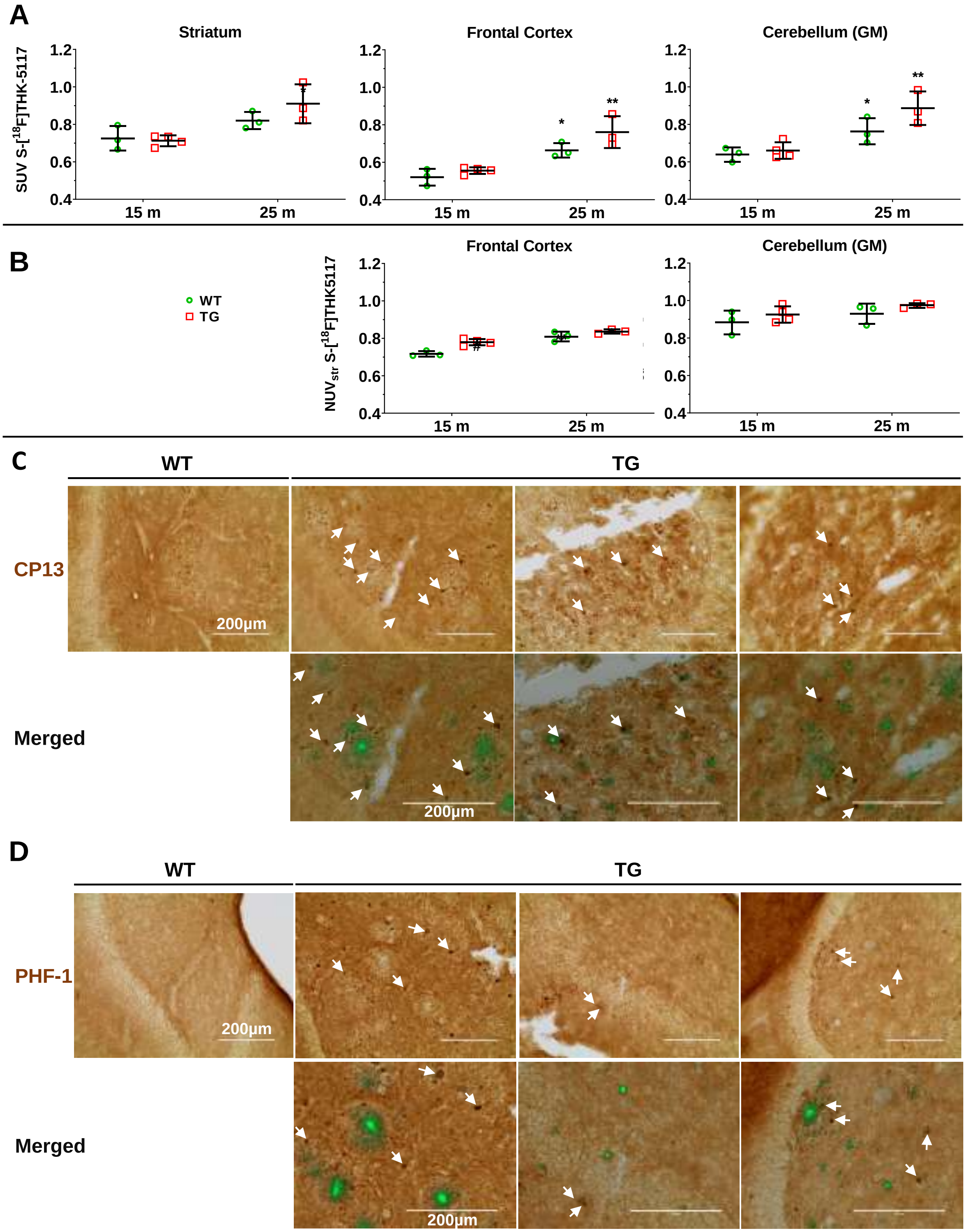

<u>Figure S8:</u> Tauopathy in the TgF344-AD rats as detected by *in vivo* by PET imaging (**A** & **B**) and immunohistochemistry (**C** & **D**). In (**A**), data are shown as SUV. In these ROI,

SUV data revealed only modest changes in [^{18}F]THK-5117 SUV driven by age without any differences between WT and TG. Expressing the data as NUV_{Str} (SUV normalised to the striatum as reference region as devoid of AD pathology) revealed genotype differences (**B**). Immunohistochemistry using CP13 (**C**) and PHF-1 (**D**) anti-Tau antibodies revealed that Tau deposition occurred mostly around amyloid plaques in the TG rats. PET data are expressed as mean ± SD of the Standard Uptake Values (***SUV*** in **A**) and Standard Uptake Values normalised to striatum (***NUV_{str}*** in **B**). * and # indicate significant difference between 15 and 25m old animals and between WT and TG respectively. */# and **/## indicate $p < 0.05$ and $p < 0.01$ respectively. PET data were analysed using a 2 way ANOVA (genotype and age) and a Sidak post-hoc test. (**C** & **D**) Scale bars represent 200µm.

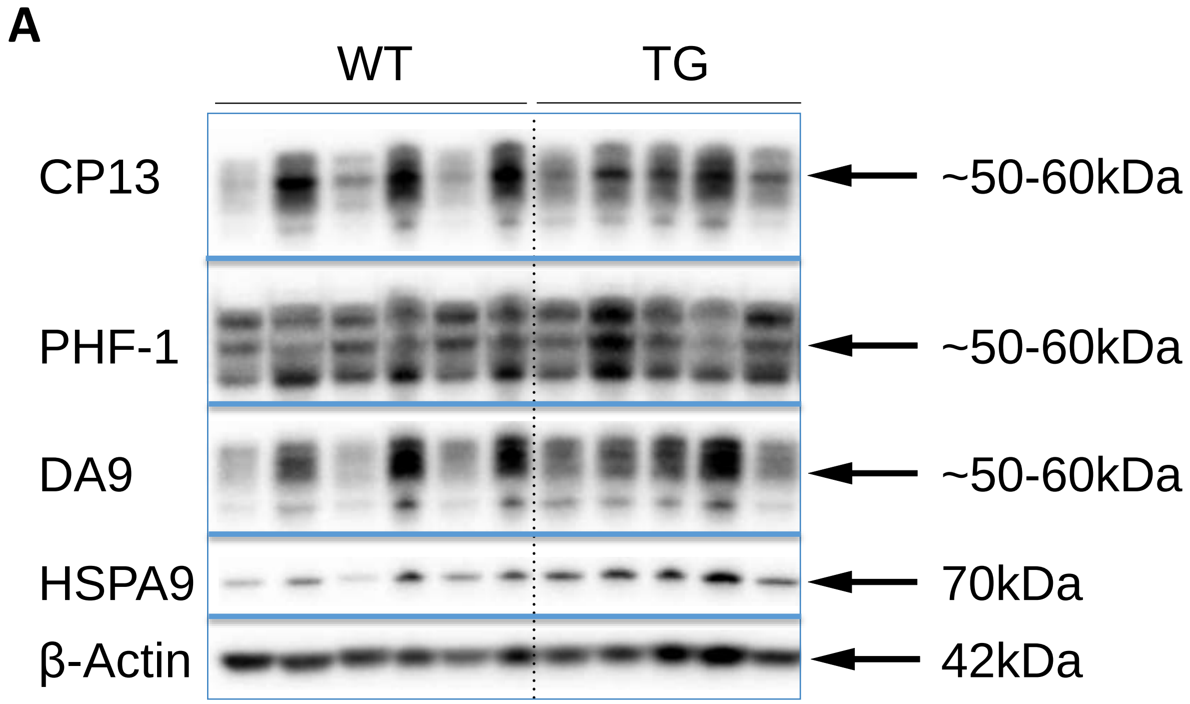

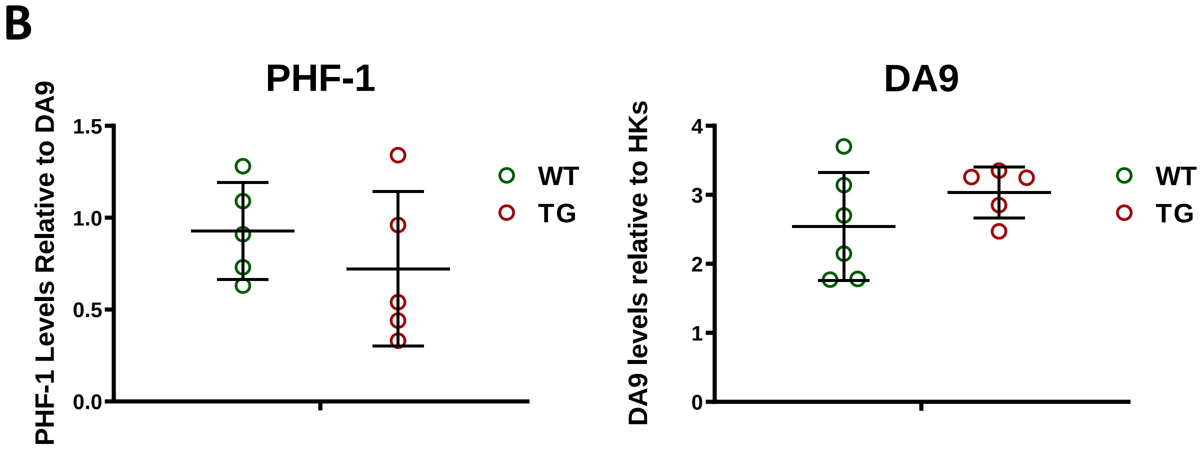

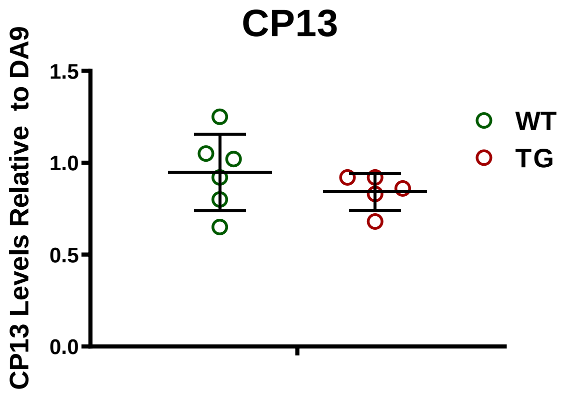

Figure S9: (**A**) representative image of a Tau protein Western blot with different antibodies. (**B**) Analysis of Tau protein Western blot on brain extract from 18 months old WT (n=6) and TG (n=5) rats. Data expressed as mean ± SD.

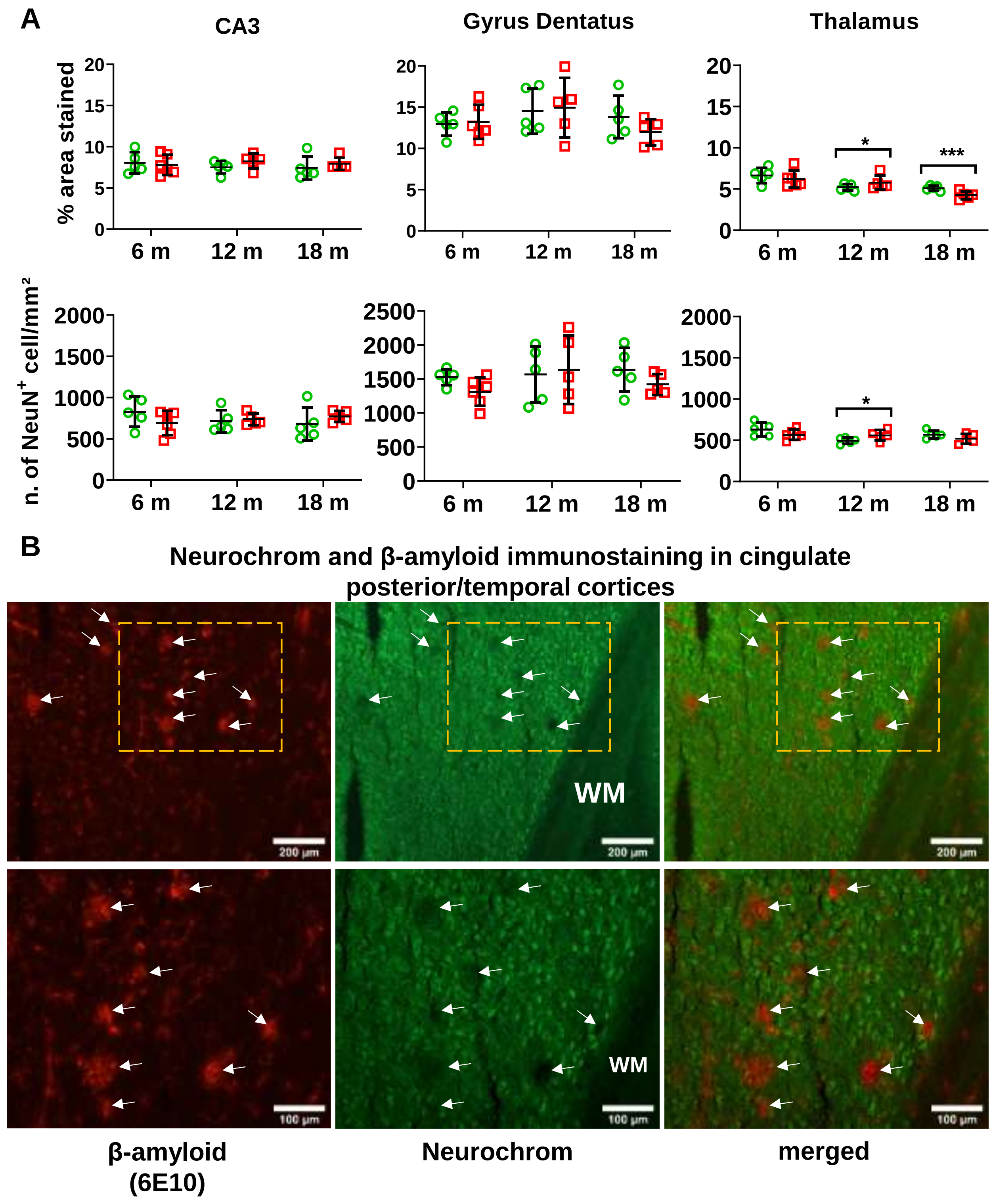

<u>Figure S10</u>: (**A**) quantification of NeuN staining by immunofluorescence expressed as percentage stained area and number of NeuN positive (***in cells/mm²***). Data are expressed as mean ± SD. (**B**) Representative micrographs of immunohistochemistry for pan-neuronal neurochrom (green) and β-amyloid (red) in the temporal/cingulate

posterior cortices of TG rats 18 months of age showing, as in the hippocampus, a clear loss of neuronal staining where β-amyloid plaques are present. (WM= white matter, Neurochrom negative). * indicates a significant difference with 6 months of age. Data were analysed using 2-way ANOVA (age as repeated factor and genotype) and a Sidak post-hoc test.

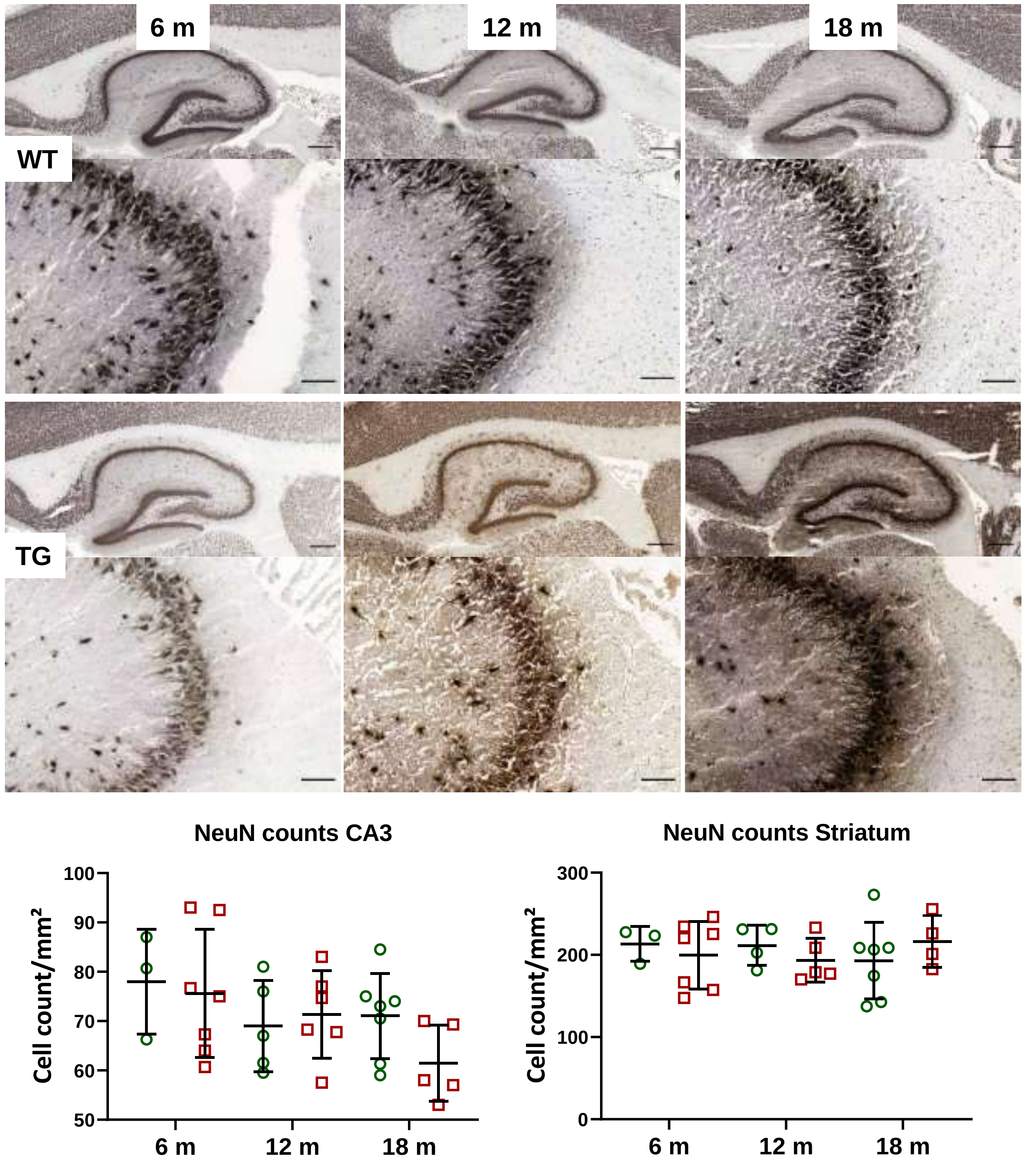

<u>Figure S11:</u> Representative micrographs of NeuN chromogenic immunohistochemistry of the hippocampus in WT and TG rats at 6, 12 and 18 months of age (top panel) and results of the manual neuronal count in the CA3 region and the striatum (as control, pathology free area; bottom panel). There was a trend of decrease with age in CA3 but not striatum. No significant differences were found between WT and TG. Data expressed as mean ± SD. Data were analysed using 2-way ANOVA (age and

genotype). Scale bars are 500µm for low magnification images and 100µm for high magnification images.

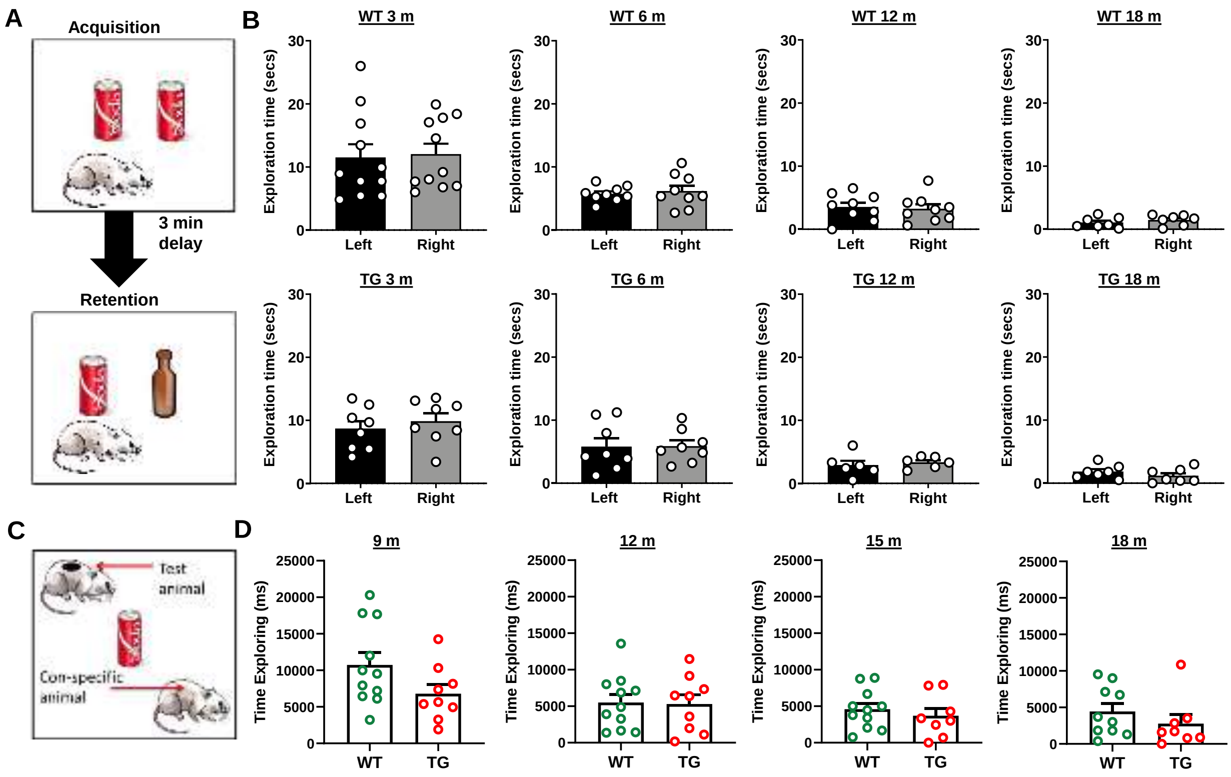

Figure S12: (**A**) Illustration of the Novel Object Recognition test set-up and (**B**) and time exploring the two identical objects in the acquisition phase in WT and TG rats at 3, 6, 12 and 18 m of age. (**C**) Illustration of the social interaction test set-up and (**D**) time spent exploring a central object. Data are shown as mean ± SEM.

Supplementary materials and methods

Behaviour: animal handling

Because both WT and TG were found to be extremely anxious, handling was carried out every day for a minimum of 3 days prior to behavioural testing and restricted to a single handler (3 weeks of handling was given for the first two time-points and was reduced to 3 days by 12 months of age). Animals were initially habituated to the test arena with littermates for 30 minutes the day prior to the 3 and 6 month tests. This became unnecessary as they were tested more frequently in the same arena. All tests were recorded without the tester in the room to allow for natural behaviour and arenas were cleaned with 70% ethanol between trials to remove any scents left by the previous rats which may alter their behaviour.

PET imaging

[^{18}F]Florbetaben and [^{18}F]ASEM

[^{18}F]Florbetaben scans were rebinned into 28 frames: 1 frame of 1 minute before injection of the tracer, then 4 frames of 10 s, 4 × 20 s, 4 × 60 s, 14 × 180 s and 1 × 120 s. The last 3 frames (43-51 min acquisition) were extracted for analysis

[^{18}F]ASEM scans were rebinned into 33 frames: 1 frame of 1 minute before injection of the tracer, then 4 frames of 10 s, 4 × 20 s, 4 × 60 s, 14 × 180 s and 6 × 120 s. The last 6 frames (49-61 min acquisition) were extracted for analysis.

Each image was corrected for randoms, scatter and attenuation, and reconstructed using a 2D OSEM (16 subsets, 2 iterations) algorithm (GE Healthcare, France) into voxels of 0.3875 × 0.3875 × 0.775mm^3. Images were analysed using PMOD (3.403, PMOD Technologies, Zurich, Switzerland, www.pmod.com). Partial volume effect correction was applied on all PET images which were co-registered to the Schiffer rat brain MRI template [1].

(S)-[^{18}F]THK5117

The radiochemical yield of (S)-[^{18}F]THK5117 was 15 ± 8%, molar activity was > 700 MBq/µmol, radiochemical purity > 98%, and shelf-life 6 hours.

The following data acquisition protocol was used: a CT scan was performed immediately prior to the PET scan for each animal to acquire attenuation correction factors. The time coincidence window was set to 3.432 ns and levels of energy discrimination to 350 keV and 650 keV. List mode data from emission scans were histogrammed into 49 dynamic frames (30 x 10s, 15 x 60s, and 4 x 300s) and emission sinograms were normalised, corrected for attenuation, scattering and radioactivity decay and

reconstructed using OSEM3D (16 subsets, 4 iterations) into 128 × 128 × 159 images with 0.776 × 0.776 × 0.796 mm^3 voxel size. Body temperature was maintained through the use of a heating pad.

The PET/CT images were pre-processed in Matlab R2017a (The MathWorks, Natick, Massachusetts, United States) with an in-house semi-automated pipeline for preclinical images that uses SPM12 (Wellcome Department of Cognitive Neurology, London, UK) pre-processing functionalities and analysis routines. Subjects were spatially normalized through a two-step registration (a rigid followed by an affine transformation) of each subject's brain CT to a template CT that was previously constructed as an average of several subjects and was aligned with an atlas T2-weighted MRI template. The combination of transformations was then applied to the PET images, which were also re-sampled to a voxel size of 0.2 x 0.2 x 0.2 mm (trilinear interpolation), matching the anatomical atlas dimensions. Region of interest analysis was performed on each subject by averaging the signal inside a slightly modified version of Schiffer rat brain atlas [1].

[^{18}F]DPA-714

[^{18}F]DPA-714 was injected intravenously as a 30s bolus (tracer & flush) in the tail vein through the use of injection pumps (syringe pump, Cole-Palmer, ref. WZ-74905-02) at the start of the PET acquisition. Animals were scanned on a Siemens Inveon® small animal PET-CT as described previously [2, 3]. The following data acquisition protocol was used: a CT scan was performed immediately prior to the PET scan for each animal to acquire attenuation correction factors. The time coincidence window was set to 3.432 ns and levels of energy discrimination to 350 keV and 650 keV. List mode data from emission scans were histogrammed into 16 dynamic frames (5 × 1 min; 5 × 2 mins; 3 × 5 mins and 3 × 10 mins) and emission sinograms were normalised, corrected for attenuation, scattering and radioactivity decay and reconstructed using OSEM3D (16 subsets, 4 iterations) into 128 × 128 × 159 images with 0.776 × 0.776 × 0.796 mm^3 voxel size. Respiration and temperature were monitored throughout the scans using a pressure sensitive pad and rectal probe (BioVet, m2m imaging corp, USA). Body temperature was maintained through the use of a heating and fan module controlled by the rectal probe via the interface of the BioVet system.

Magnetic Resonance Spectroscopy Acquisition and Analysis

Structural MRI (Fast Low Angle SHot: 9 slices, 2 averages, 0.5 mm inter-slice distance) and MRS were carried out using a 7 Tesla horizontal-bore magnet (Agilent Technologies, Oxford Industrial Park, Yarnton, Oxford, UK) interfaced to a Bruker AVANCE III spectrometer (Bruker Spectrospin, Coventry, UK). In brief, FASTMAP [4] was used to shim a 10 mm^3 region encompassing the planned voxels. The water linewidth at full-height half-maximum (LW) was estimated and if below 20Hz, the water-suppressed acquisition was run. Re-shimming was performed twice if LW > 20Hz. The NAA LW was measured in all the spectra to act as a quality metric. These data are reported in Table S6. The

VAPOR method was used for water suppression [5] and a PRESS (Point REsolved SpectroScopy) [6] sequence (repetition time 2500 ms, echo-time 20 ms) was carried out. For large ROIs (hippocampus and thalamus), 128 averages were acquired and for smaller ROIs (right hippocampus, hypothalamus and cortex) 512 averages were acquired for sufficient signal-to-noise. Spectra were quantified in the time domain using the QUEST routine [7]in jMRUI [8] as previously described [2]. A metabolite basis-set was simulated using NMRScope. Metabolites in the basis-set included NAA (N-acetylaspartate), glutamate, *myo*-inositol, total-creatine (creatine and phosphocreatine), taurine, tCho (choline, phosphorylcholine, glycerophosphorylcholine), glutamine, γ-aminobutyric acid (GABA), and *scyllo*-inositol. Peaks at 0.9, 1.3 ppm were included to model macro-molecules and a peak at 3.75 ppm was added to account for α-amino acid protons that have been previously observed in brain MRS of rodents and are not accounted for by glutamate and glutamine [9].

Immunohistochemistry

Animals were culled by isoflurane overdose confirmed by cervical dislocation. The brains were collected, snap frozen using isopentane on dry ice and stored at -80°C. Twenty-five sets (slides) of 4 sagittal brain sections (20µm thick) were taken on SuperFrost Plus glass slides using a cryostat (Leica CM3050s, Leica Biosystems Nussloch GmbH, Germany) from 1, 1.62, 2.24 and 2.86 mm lateral of Bregma and stored at -80°C.

Immunofluorescence for GFAP & CD11b, NeuN, Neurochrom and β-amyloid (6E10)

Sections were allowed to defrost and dry at room temperature (10~20min) and then fixed with 4% paraformaldehyde for 10 min before being washed (6 × 5min) in phosphate buffered saline (PBS) and incubated for 30 min in 2% normal donkey serum and 0.1% Triton X-100 in PBS to permeabilise and block non-specific binding. Primary antibody incubation was carried out overnight at 4°C with one of the following primary antibodies in 2% normal donkey serum and 0.1% Triton X-100 in PBS: mouse anti-rat CD11b (AbD Serotec (MCA275G) 1:1000); rabbit anti-rat GFAP (DAKO (Z0334) 1:1000); mouse anti-human 6E10 amyloid (BioLegend (803001) 1:1000); rabbit anti-rat NeuN (Abcam (ab177487), 1:500); rabbit anti-rat Neurochrom (Millipore (ABN2300) 1:100). Following incubation in primary antibody, PBS washes were repeated (3 × 10min) and incubated with one of the following secondary antibodies (Molecular Probes, Invitrogen) was carried out: Alexa Fluor 594nm Donkey anti-mouse IgG 1:500 (for CD11b and 6E10); Alexa Fluor 594nm Donkey anti-rabbit IgG 1:500 (for NeuN); Alexa Fluor 488nm Donkey anti-rat IgG 1:500 (for GFAP and Neurochrom). Double staining was carried out for CD11b+GFAP and 6E10+neurochrom.

Image analysis was performed using Fiji [10]. For amyloid quantification the following steps were used: original snapshot were duplicated in 8bit, a mask with radius = 100 and mask = 0.60, blackbackground = True was set, and converted to an 8-bit mask, fill holes and erode was then applied,

images were calibrated in µm and particles were analysed with the following settings: size = 750-25000 µm, circularity = 0.22-0.90 and "clear summarize add". For NeuN analysis the following parameters were used: snapshots were converted in 8-bit images and calibrated in µm and duplicated, an Unsharp Mask filter (radius = 100 and mask = 0.60) and an Otsu threshold were applied, followed by a binary watershed filter, Analyse Particles was then used (particle size in pixels 25-5000, circularity 0.25-1.00). For GFAP and CD11b, images were calibrated in µm and, for the hippocampus cropped if necessary to remove the corpus callosum and potential part of the cortex taken in the snapshot, images were then converted to 8bit and thresholded visually to remove background or staining artefact to keep only cells visible, the total staining area and percentage stained area were then measured.

Chromogenic NeuN immunohistochemistry and analysis

Sagittal sections were washed 3 × 5 minutes with PBS between stages and agitated on a shaker plate throughout incubation. Sections were incubated in a 0.1M citrate 6.0pH antigen retrieval buffer for 20 minutes at 80°C in a water bath. Sections were then washed followed by blocking and permeabilising in 5% normal goat serum and 0.05% Triton X-100 in PBS for one hour at room temperature. After blocking, rabbit anti-NeuN (AB177487, Abcam) was added to blocking solution (1:500) and incubated overnight at room temperature. The next day, sections were washed then incubated in Vector anti-rabbit IgG (1:1000 blocking solution) for 1 hour, then agitated with Avidin-Biotin (both 1:500 in PBS) solution for 30 minutes. Finally, the sections were developed using a Vector DAB HRP substrate kit for 10 minutes.

Images of full slides were taken using a 3D Histec Pannoramic 250 slide scanner. NeuN analysis was conducted using 3D Histec CaseViewer software. To consistently count NeuN+ neurons while minimising false positive labelling such as from cellular debris, manual cell counts were conducted in 303 × 455µm regions of interest in hippocampus and striatum [11].

Cell counts per region of interest, per slice, per animal were taken along the medio-lateral axis through hippocampus and striatum. To ensure between group comparisons were fair and consistent, we stained every other section with neutral red to label Nissl bodies. These sections were used to allocate sections to their distance from the mid-sagittal plane, with reference to the Paxinos and Watson rat brain atlas [12]. Sections were coded as being from 1 to 5mm lateral based on the anatomical shape of the hippocampus and striatum. Due to anatomical variation along this axis, only sections from 2mm to 4mm lateral of the mid-sagittal plane were included in statistical analyses. The mean number of NeuN+ neurons per region of interest per animal was calculated and summary group statistics and factorial analyses conducted on these data. A total of 40,538 NeuN+ neurons were manually counted (*n* rats per group: WT 6 m: 3, TG 6 m: 7, WT 12 m: 5, TG 12 m: 6, WT 18 m: 7, TG 18 m: 5).

Tau Western blot analysis

Approximately 100mg of cortex tissue was homogenised in 1ml cold RIPA buffer as previously described Salih et al. (2012) [13], [1% (v/v) Triton X-100, 1% (w/v) sodium deoxycholate, 0.1% (w/v) SDS, 0.15 M NaCl, 20 mM Tris HCl (pH 7.4), 2 mM EDTA (pH 8.0), 50 mM sodium fluoride, 40 mM β-glycerophosphate, 1 mM EGTA (pH 8.0), 1 mM EGTA (pH 8.0), 2 mM sodium orthovanadate; and (adding just before use), 1 mM PMSF (in 100% ethanol), one Protease inhibitor tablet (11 836 153 001; Roche), Sigma phosphatase inhibitor cocktail 2 (× 100 dilution), Sigma phosphatase inhibitor cocktail 3 (× 100 dilution) by using a Sonicator (Branson sonifier, 450). Next, homogenates were centrifuged at 13,000 RPM for 10 min at 4ºC, supernatant protein concentration was measured using a Bradford assay and samples stored at -80°C.

Samples, mixed with dye, were boiled in 95ºC, 10 min (Tau samples) or 2 min (ordinary samples) before storage at -20ºC.

The SDS-PAGE gel, composed by 4% stacking gel and 10-15% resolving gel, was poured into Novex 1.5mm gel casting trays (Invitrogen). 50µg protein of each sample was loaded. The stated amount of protein of each sample was normalized to a final volume of 15 µl by cold 1 × loading dye. Samples were run at 100V and 130V through the stacking gels and resolving gels, respectively. Then, proteins were transferred to nitrocellulose membrane (0.45 µM, BIO-RAD) at 30V, room temperature overnight. Membranes were blocked in 5% fat-free milk in 0.1%Tween-20 in TBS (TBST) for 1 hour and incubated with primary antibody (see details in Table S7) for 2 h at room temperature, signals were visualized by secondary antibody incubation (1:10,000) in blocking buffer followed by Clarity™ Western ECL Blotting Substrates (BIO-RAD) development. Imaging was performed using the BIO-RAD ChemiDoc MP imaging system. The western blot images were analysed by Image Lab software (BIO-RAD).

Supplementary information

Table S1: University of Manchester cohort. Animals used in the study.

		Age (months)			Dead/Excluded for *ex vivo*	TOTAL
		≤6 m	12 m	*18 m*		
WT	*In vivo* imaging & behaviour*	**10**	*9***		3[†]	**10**
	Ex vivo only	5	5	4	4	18
TG	*In vivo* imaging & behaviour*	**11**	10	*9***	4[#]	**11**
	Ex vivo only	8	6	2	2	18
					GRAND TOTAL:	57

* Animals used for *in vivo* imaging and behaviour were monitored longitudinally, i.e. animals were kept for 18-19 months. ** Animals used for the *in vivo* longitudinal study were used for *ex vivo* analysis for the 18-19 months time-point. [†] 1 animal died before the 18 months time-point, 2 were excluded after post-mortem examination at 18 months. [#] 1 animal died before the 12 months time-point, 1 animal died before the 18 months time-point, 2 were excluded after post-mortem examination at 18 months. Criteria for exclusion: presence at post-mortem examination of stroke, subarachnoid haemorrhage, intracerebral haemorrhage or tumours; please see Table S4 for more details.

Table S2: Age of the WT and TG rats (in days) and average weight (in grams) of rats when scanned with [^{18}F]DPA-714 at each time-points (University of Manchester cohort). Data are expressed as mean±SD. N numbers are indicated in brackets.

	6 months		12 months		18 months	
	WT (10)	TG (11)	WT (9)	TG (10)	WT (9)	TG (9)
Age (days)	191±4	189±10	369±11	363±10	557±8	558±13
Average weight	391±19g	423±22g	464±24g	493±20g	480±27g	478±31g

Table S3: Age of the WT and TG rats (in days) and average weight (in grams) when scanned with [^{18}F]Florbetaben and [^{18}F]ASEM at each time-points (University of Tours cohort). Data are expressed as mean±SD (n = 8 per group).

		6 months		12 months		18 months	
		WT	TG	WT	TG	WT	TG
Age (days)	[^{18}F]florbetaben	194±2	198±5	369±16	361±12	540±17	537±5
	[^{18}F]ASEM	191±27	197±21	374±15	371±15	527±14	536±9
Average weight		414±9g	407±9g	472±12g	478±8g	460±14g	471±12g

Table S4: causes of mortality or exclusions in the University of Manchester cohort.

	**Overnight death, no identifiable cause**	**SAH**	**ICH**	**Stroke**	**Tumours (lungs & liver) & hypertrophic spleen**
**WT**	1 (**392**)	2 (**541**†, 605†)	0	2 (**513**, **590**†)	2 (589, 605)
**TG**	1 (**179**†)	0	2 (**538**, **539**†)	0	3 (**357**, **540**†, 605†)

Age of death (in days) are indicated in brackets; ages in **bold** indicate animals that **died** or were **culled** due to illness before reaching the planned time-point; other animals were excluded and their cause of exclusion was identified at the planned time-points of euthanasia. † Indicates animals that were used for imaging and behaviour and died before reaching the end point of the study or were excluded at post-mortem examination at 18-19 months. SAH: subarachnoid haemorrhage. ICH: intracerebral haemorrhage.

Table S5: number of animals used for each immunohistochemistry quantitative measures.

	6 months		12 months		18 months	
Antigen	WT	TG	WT	TG	WT	TG
CD11b	4	5	5	5	8	9**
GFAP	4*	5	5	5	8	9
NeuN (fluo.)	5	6	5	5	5	5
NeuN (chromog.)	3	7	5	6	7	5
Aβ (6E10)	ND	6	ND	7	ND	6

***** One WT 6 m was identified as an outlier and excluded for the GFAP quantification in the temporal/posterior cingulate cortex and thalamus. ****** One TG 18 m was identified as an outlier and excluded from the CD11b quantification in the thalamus. **ND**: no Aβ detected in WT rats, not quantified.

Table S6: NAA linewidth (mean±SD) for each brain region.

**Brain Region**	**NAA LW**
All regions	16.2±6.5
Full hippocampus	19.0±8.7
Right hippocampus	18.3±5.3
Thalamus	16.1±6.1
Hypothalamus	13.6±5.0
Cortex	14.2±4.7

Table S7: Antibodies used for Tau immunohistochemistry and Western blot analysis.

Antibody	Company	Species	Concentration	Blocking solution
PHF-1	Gift from Prof. Peter Davies	Mouse monoclonal	1:1000 (WB); 1:600 (IHC)	5% powdered milk in TBS (with 0.02% Azide, for WB); 2.5% Normal Horse Serum in PBS (IHC)
CP13	Gift from Prof. Peter Davies	Mouse monoclonal	1:1000 (WB); 1:600 (IHC)	5% powdered milk in TBS (with 0.02% Azide, for WB); 2.5% Normal Horse Serum in PBS (IHC)
DA9	Gift from Prof. Peter Davies	Mouse monoclonal	1:5000 (WB)	5% powdered milk in TBS (with 0.02% Azide, for WB)
HSPA9	Thermofisher, MA1-094	Mouse monoclonal	1:1000 (WB)	5% BSA in TBS (with 0.02% Azide, for WB)
β-Actin	Abcam, ab8227	Rabbit Polyclonal	1:20000 (WB)	5% BSA in TBS (with 0.02% Azide, for WB)
AT8	Thermofisher, MN1020	Mouse monoclonal	1:600 (IHC)	2.5% Normal Horse Serum in PBS (IHC)

References

1. Schiffer WK, Mirrione MM, Dewey SL. Optimizing experimental protocols for quantitative behavioral imaging with 18F-FDG in rodents. J Nucl Med. 2007; 48: 277-87.
2. Chaney A, Bauer M, Bochicchio D, Smigova A, Kassiou M, Davies KE, et al. Longitudinal investigation of neuroinflammation and metabolite profiles in the APPswe xPS1Deltae9 transgenic mouse model of Alzheimer's disease. J Neurochem. 2018; 144: 318-35.
3. Sridharan S, Lepelletier FX, Trigg W, Banister S, Reekie T, Kassiou M, et al. Comparative Evaluation of Three TSPO PET Radiotracers in a LPS-Induced Model of Mild Neuroinflammation in Rats. Mol Imaging Biol. 2017; 19: 77-89.
4. Gruetter R. Automatic, localized in vivo adjustment of all first- and second-order shim coils. Magn Reson Med. 1993; 29: 804-11.
5. Griffey RH, Flamig DP. VAPOR for Solvent-Supressed, Short-Echo, Volume-Localized Proton Spectroscopy. Journal of Magnetic Resonance. 1990; 88: 161-6.
6. Bottomley PA. Spatial localization in NMR spectroscopy in vivo. Annals of the New York Academy of Sciences. 1987; 508: 333-48.
7. Ratiney H, Sdika M, Coenradie Y, Cavassila S, van Ormondt D, Graveron-Demilly D. Time-domain semi-parametric estimation based on a metabolite basis set. NMR Biomed. 2005; 18: 1-13.
8. Stefan D, Di Cesare F, Andrasescu, A. Popa E, Lazariev A, Vescovo E, et al. Quantitation of magnetic resonance spectroscopy signals: The jMRUI software package. Measurement Science and Technology. 2009; 20: 104035.
9. Forster D, Davies K, Williams S. Magnetic resonance spectroscopy in vivo of neurochemicals in a transgenic model of Alzheimer's disease: a longitudinal study of

metabolites, relaxation time, and behavioral analysis in TASTPM and wild-type mice. Magn Reson Med. 2013; 69: 944-55.
10. Schindelin J, Arganda-Carreras I, Frise E, Kaynig V, Longair M, Pietzsch T, et al. Fiji: an open-source platform for biological-image analysis. Nat Methods. 2012; 9: 676-82.
11. Ahn JH, Choi JH, Park JH, Kim IH, Cho JH, Lee JC, et al. Long-Term Exercise Improves Memory Deficits via Restoration of Myelin and Microvessel Damage, and Enhancement of Neurogenesis in the Aged Gerbil Hippocampus After Ischemic Stroke. Neurorehabil Neural Repair. 2016; 30: 894-905.
12. Paxinos G, Watson C. The Rat Brain in Stereotaxic Coordinates. 6 ed: Academic Press; 2007.
13. Salih DA, Rashid AJ, Colas D, de la Torre-Ubieta L, Zhu RP, Morgan AA, et al. FoxO6 regulates memory consolidation and synaptic function. Genes Dev. 2012; 26: 2780-801.
